# Insight into the fission mechanism by quantitative characterization of Drp1 protein distribution in the living cell

**DOI:** 10.1038/s41598-018-26578-z

**Published:** 2018-05-25

**Authors:** Bernadeta Maria Michalska, Karina Kwapiszewska, Joanna Szczepanowska, Tomasz Kalwarczyk, Paulina Patalas-Krawczyk, Krzysztof Szczepański, Robert Hołyst, Jerzy Duszyński, Jędrzej Szymański

**Affiliations:** 1 Laboratory of Bioenergetics and Biomembranes, Department of Biochemistry, Nencki Institute of Experimental Biology, Polish Academy of Sciences, 3 Pasteur Str, 02-093 Warsaw, Poland; 2 Institute of Physical Chemistry, Polish Academy of Sciences, Kasprzaka 44/52, 01-224 Warsaw, Poland

## Abstract

One of the main players in the process of mitochondrial fragmentation is dynamin-related protein 1 (Drp1), which assembles into a helical ring-like structure on the mitochondria and facilitates fission. The fission mechanism is still poorly understood and detailed information concerning oligomeric form of Drp1, its cellular distribution and the size of the fission complex is missing. To estimate oligomeric forms of Drp1 in the cytoplasm and on the mitochondria, we performed a quantitative analysis of Drp1 diffusion and distribution in gene-edited HeLa cell lines. This paper provides an insight into the fission mechanism based on the quantitative description of Drp1 cellular distribution. We found that approximately half of the endogenous GFP-Drp1 pool remained in the cytoplasm, predominantly in a tetrameric form, at a concentration of 28 ± 9 nM. The Drp1 mitochondrial pool included many different oligomeric states with equilibrium distributions that could be described by isodesmic supramolecular polymerization with a K_d_ of 31 ± 10 nM. We estimated the average number of Drp1 molecules forming the functional fission complex to be approximately 100, representing not more than 14% of all Drp1 oligomers. We showed that the upregulated fission induced by niclosamide is accompanied by an increase in the number of large Drp1 oligomers.

## Introduction

Mitochondria form a highly complex and dynamic structure in the cell and undergo continuous reshaping by fusion and fission. Their main role is the production of ATP, but they also control calcium buffering^1^ and other cellular processes, which depend on the ability of mitochondria to dynamically change their shape and integrity^2^. Fission enables the release of mitochondrial components to the cytoplasm and is responsible for the fragmentation of the mitochondrial network, which is important in many cellular processes such as mitophagy^3,4^, the induction of apoptosis^5^, the transport of mitochondria along the cytoskeleton^6^, the distribution of mtDNA in the mitochondrial network^7,8^ and the equal distribution of mitochondria to daughter cells during cell division^9^. Defects in the fission machinery can lead to several diseases such as diabetes^10^ and to several neurodegenerative disorders such as Alzheimer’s disease^11^, Parkinson’s disease^12^, Huntington’s disease^13^ and glaucoma^14^.

One of the major players in the fission process is dynamin-related protein 1 (Drp1), a cytosolic GTPase with a propensity for oligomerization. The recruitment of Drp1 from the cytoplasm to the mitochondria is mediated by several outer mitochondrial membrane (OMM) proteins, including Mff, MiD49, MiD51, and Fis1^15–17^, and by the mitochondria-specific lipid cardiolipin^18^. Recent reports indicate that Drp1 maintains an equilibrium between its cytosolic and mitochondrial fractions^19^, however more detailed description of subcellular Drp1 distribution is missing. Dynamic rearrangements between mitochondrial Drp1 oligomers allow for their progressive maturation into ring-like structures wrapping around mitochondria^19^. Their size has been a subject of several studies and resulted in estimates ranging from 30–50 nm (ring composed of 16–20 Drp1 monomers)^20,21^ to Drp1 rings of 130–150 nm^22,23^ (formed by 48 Drp1 tetramers)^22^ the latter additionally shown to constrict during fission to around 75–78 nm upon GTP addition. Those structures can perform fission if additional signals occur at the potential fission sites. Several such signals have been identified, which involve actin^19,24^ and the endoplasmic reticulum (ER)^25^. The ER encircles the mitochondrion prior to fission and is responsible for the initial reduction in its diameter. Actin filaments facilitate the assembly of the productive fission complex and stimulate Drp1 GTPase activity, enabling the generation of the constrictive force by the Drp1 ring. Recently, a new mitochondrial fission machinery component has been discovered, namely, dynamin-2 (Dyn2). Dyn2 has been proposed to act during the last step of mitochondrial fission, and its role is to complete division by the final mitochondrial membrane constriction, which is preceded by Drp1-mediated constriction^26^. The list of components involved in the mitochondrial fission event is expanding, and the sequence of events in this process is under investigation.

Drp1 is recruited to mitochondria from the cytoplasm, where the oligomeric form of Drp1 is still being elucidated, however *in vitro* studies suggest that Drp1 in the cytosol form dimers^27,28^, tetramers^29,30^ or exist in dimer-tetramer equilibrium^22^. Several studies have reported on the specificity of different oligomeric forms of Drp1 for MiD or Mff; however, some studies report conflicting results concerning the exact oligomeric forms of Drp1 involved in the interactions^31,32^. The affinity of Drp1 to mitochondrial receptors is also regulated at the level of Drp1, which is present in several isoforms^33,34^ (Supplementary Table S2) and additionally can undergo several posttranslational modifications^35^. The oligomers formed by disparate Drp1 isoforms differ with respect to size, preferred curvature, and GTPase activity, which can directly affect their ability to fragment the mitochondrial network^33^. Many studies in which oligomeric forms of Drp1 were examined were based on *in vitro* studies performed with selected specific Drp1 isoform obtained as a recombinant protein in which additional changes could be introduced by site directed mutagenesis^20,22,27^. However in the cellular environment the oligomeric form of Drp1 could depend on relative abundance of different isoforms or postranslational modifications of Drp1, which shows the need for the experimental estimate of the Drp1 oligomeric form obtained using live cell studies on the gene edited cell lines.

The activity of Drp1 in mitochondrial fission is regulated at many different levels and our understanding of the mechanism of Drp1 fission remains limited. Detailed description of the subcellular distribution of Drp1 could help to better understand this process. In the present work we estimated the oligomeric form of Drp1 from the diffusion coefficient measured in the cytoplasm of the gene edited GFP-Drp1 HeLa cell lines. The obtained results are interpreted within the length-scale dependent viscosity model (LDVM)^36^ which can be used to describe the viscosity of various complex environments including the cytoplasm of the cell. Additionally, using single molecule fluorescence method, we provide also a quantitative description of the distribution of the mitochondrial Drp1 oligomers and estimate the size of the productive fission complex. We propose a mechanism in which fission can be regulated by shifting the GFP-Drp1 oligomer distribution toward larger oligomers, thus increasing the fraction of fission-competent Drp1 oligomers.

## Results

### The viscosity of cytoplasm in HeLa cells described by LDVM

The size of a macromolecule given as a hydrodynamic radius, *r*_*p*_, can be estimated from the Einstein-Stokes relation,

1 $$D=kT/(6\pi \eta {r}_{p}),$$

**Figure 1:**
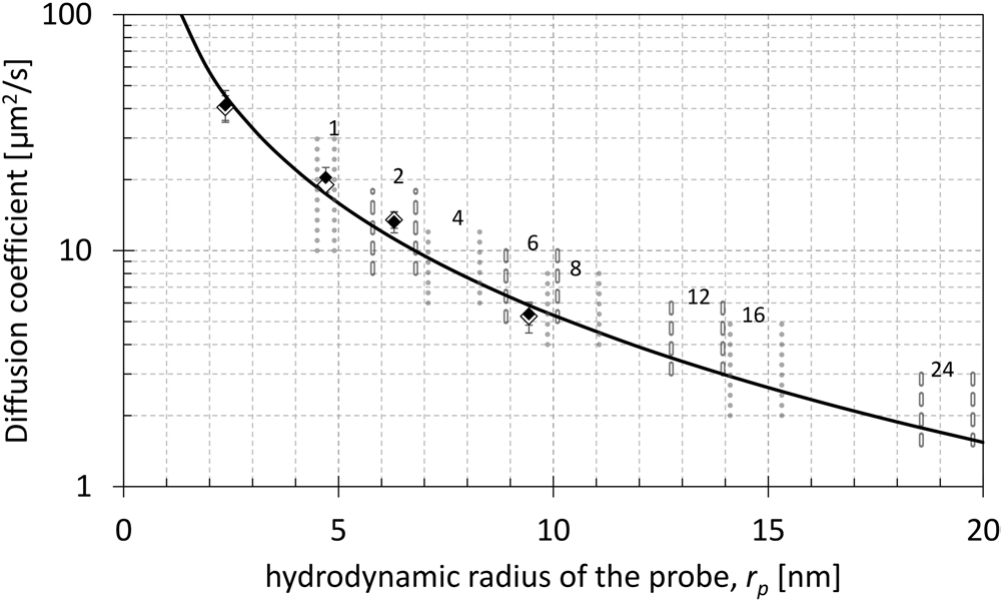
Predicted values of diffusion coefficients (solid black line) based on LDVM as a function of the size r_p_ calculated for different Drp1 oligomers. The size ranges for different oligomers are indicated by vertical dotted and dashed lines, with the number of Drp1 molecules forming an oligomer shown at the top. The experimental values of diffusion coefficients measured for test probes of increasing size, including GFP (r_p_ = 2.4 nm) GFP-Drp1 K668E (monomeric Drp1, r_p_ = 4.7 nm), GFP-Drp1 G363D (dimeric Drp1, r_p_ = 6.7 nm) and GFP-ferritin (r_p_ = 9.4 nm) are shown as full black diamonds (for HeLa wt) and empty diamonds (for HeLa Drp1 KO).

**Figure 2:**
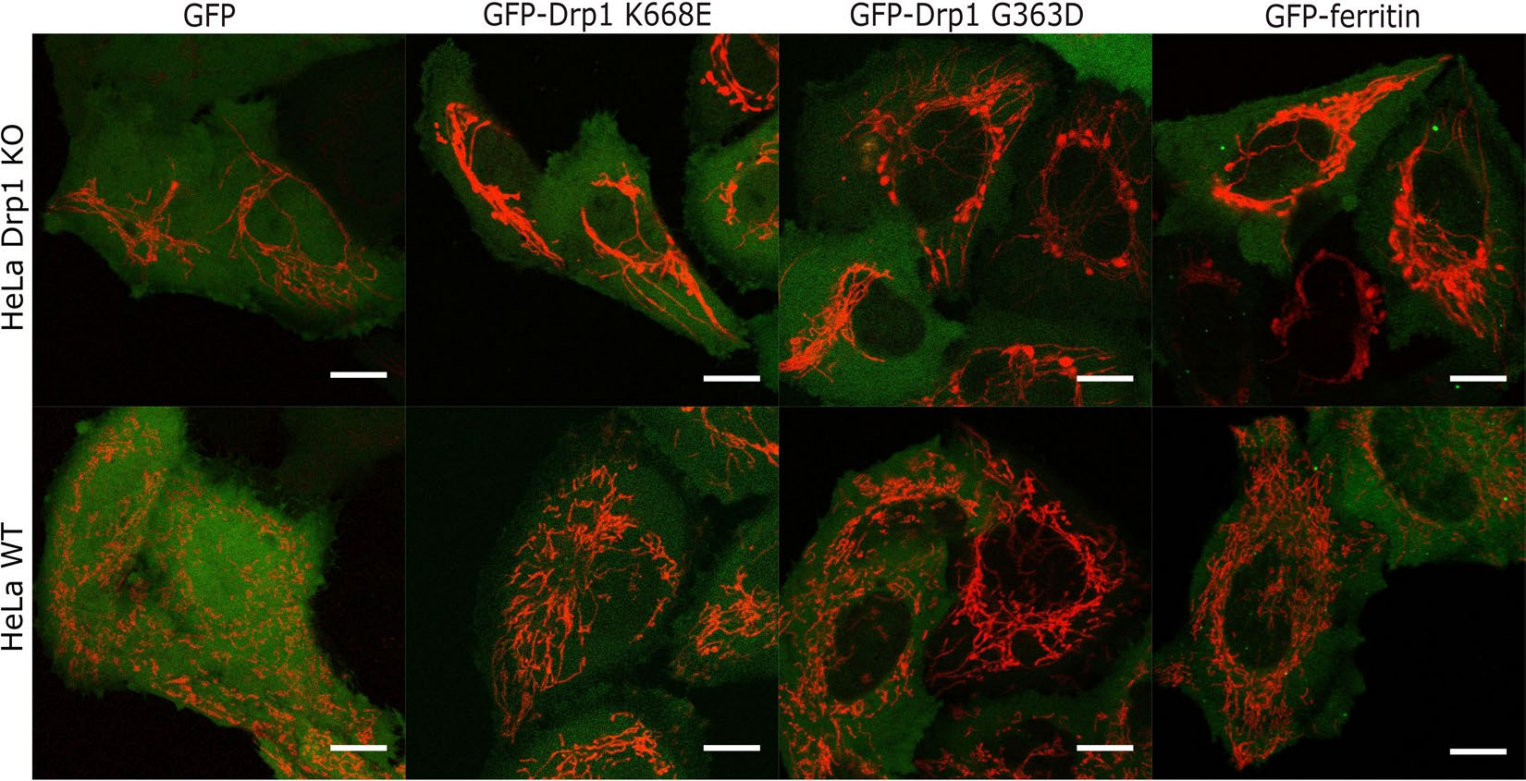
Expression of GFP alone, GFP-Drp1 mutants (K668E corresponding to monomeric Drp1 and G363D corresponding to dimeric Drp1) and GFP-ferritin in HeLa Drp1 KO and in non-modified HeLa (HeLa wt) cell lines. These constructs were used for the calibration of LDVM. The lack of expression of Drp1 in the HeLa Drp1 KO cells resulted in an elongated mitochondrial network compared to the mitochondrial morphology in HeLa wt. Mitochondria are shown in red (transfection with Mito-mNeptune), and the expression of GFP-tagged constructs is shown in green. Scale bar, 10 μm.

In which *D* denotes the diffusion coefficient, *k* the Boltzmann constant, *T* the absolute temperature and *η* the viscosity. One of the goals of this work was to estimate the size and hence the oligomeric form of Drp1 from its diffusion coefficient measured in the cytoplasm of the living cells at the temperature of 37 °C and taking into account the estimated value of the cytoplasmic viscosity. Although the cytoplasm of a cell consists largely of water, it has different properties from those of simple aqueous solution. For complex fluids, the viscosity, *η*, experienced by different probes is not constant but depends on the size, *r*_*p*_, of the probe, a phenomenon referred to as the length scale-dependent viscosity model (LDVM)^37–39^. The LDVM can also provide an estimate of the effective viscosity sensed by probes of different sizes diffusing in the cytoplasm of the cell^36^. One of our goals was to adapt the LDVM to cell lines studied in the present work. To estimate the cytoplasmic viscosity of the HeLa cell line we have measured using Fluorescence Correlation Spectroscopy (FCS) the diffusion coefficients for test probes with known sizes ranging from 2.4 to 9.4 nm (see Methods), including macromolecules of GFP, Drp1 monomer (GFP-Drp1 K668E), Drp1 dimer (GFP-Drp1 G363D) and GFP-ferritin (FTH1). In order to assess the influence of the endogenous Drp1 present in the non-modified HeLa (HeLa wt) we have performed also measurements in the HeLa Drp1 knockout (KO) cell line, in which we could study the diffusion of different variants of GFP-Drp1 introduced by transfection without background of endogenous Drp1. We did not observe differences between the measured diffusion coefficients obtained in two cell lines (Fig. 1) indicating that the influence of the endogenous Drp1 is negligible for the studied GFP-Drp1 mutants. The lack of interactions between endogenous Drp1 and studied probes is additionally confirmed by the confocal imaging showing the expression of the diffusion test probes in HeLa Kyoto wild type (wt) and HeLa Kyoto Drp1 knockout (KO) (Fig. 2). In all cases (Fig. 2), a uniform distribution of fluorescence throughout the cytoplasm is observed, indicating that no high-order oligomerization of Drp1 occurs for the selected Drp1 mutants. Drp1 is not enriched at the mitochondria, indicating that oligomerization is necessary for the efficient binding of Drp1 to OMM receptors (OMMRs).

The diffusion coefficients obtained for the test probes were used to adjust the LDVM parameters for studied cell lines (see Methods). With the established LDVM we could predict the diffusion coefficient in the cytosol of studied cell line for the probe of any given size, *r*_*p*_. For each oligomer, we predicted diffusion coefficient in the cytoplasm (full black line in Fig. 1), using its calculated size to scale the cellular viscosity according to LDVM (see Methods and Supplementary Table 1).

### Diffusion, oligomeric form and concentration of Drp1 in HeLa cells

**Figure 3:**
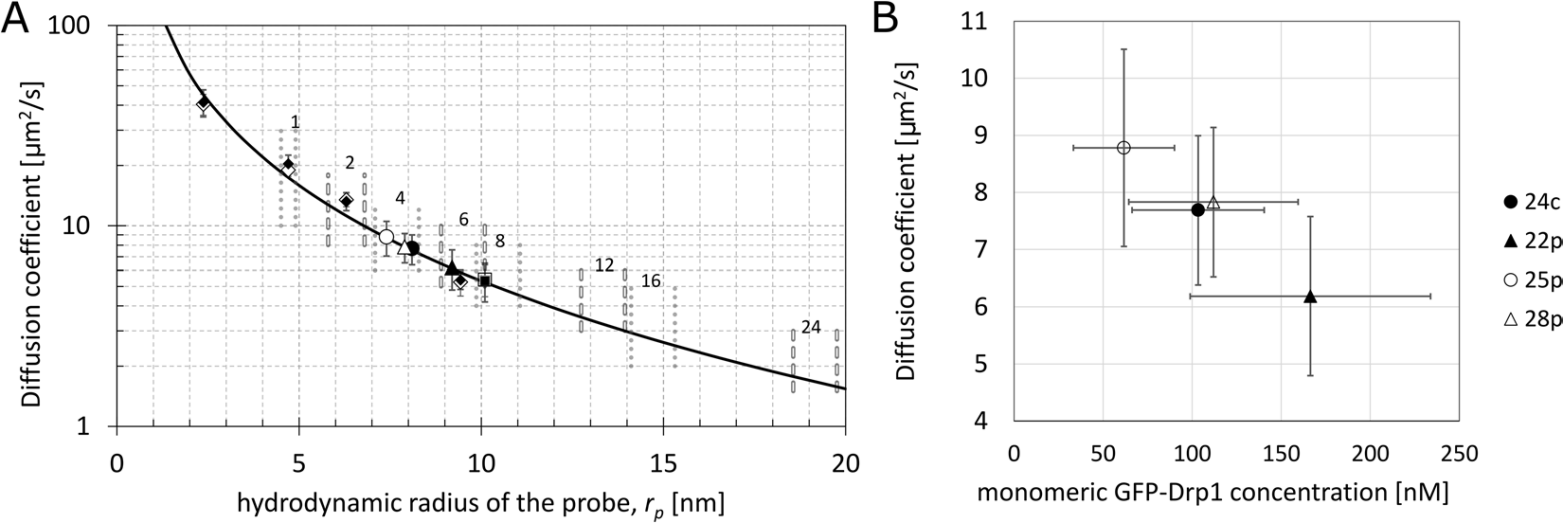
Diffusion coefficients for GFP-Drp1 obtained from FCS measurements. (**A**) Diffusion coefficients presented as a function of the hydrodynamic radius of the probe in LDVM for GFP-Drp1 in stable HeLa cell lines (full black circles 24c, full black triangles 22p, empty circles 25p, empty triangles 28p) and for GFP-Drp1 isoform 1 in transfected HeLa cells (full black squares for HeLa wt, empty squares for HeLa Drp1 KO). The average values of the diffusion coefficients measured in the cytoplasm in the stable cell lines were 6.2 ± 1.4, 7.7 ± 1.3, 7.8 ± 1.3 and 8.8 ± 1.7 μm^2^ × s^−1^ for the 22p, 24c, 28p and 25p cell lines, respectively. Error bars represent standard deviation. (**B**) Graph representing the correlation between the diffusion coefficients of GFP-Drp1 in stable HeLa cell lines and the concentration of GFP-Drp1 (expressed as monomeric GFP-Drp1 concentration, which is four-fold higher than the tetramer concentration). The average values of the GFP-Drp1 tetramer concentrations calculated based on the autocorrelation function (ACF) from FCS were 26 ± 9, 42 ± 17, 15 ± 7, and 28 ± 12 nM for the 24c, 22p, 25p, and 28p cell lines, respectively. Error bars represent standard deviation.

Predicted values of diffusion coefficients (Fig. 1) were used to determine the oligomeric form of GFP-Drp1 molecule in the cytoplasm of living cells. In order to achieve this goal we measured the diffusion coefficients of GFP-Drp1 in the HeLa Kyoto cell line transfected with a plasmid coding for GFP-Drp1 isoform 1 and in four stable cell lines, which were obtained using CRISPR/Cas9 gene editing by placing an insert of mEGFP upstream of exon 1 of the DNM1L gene encoding protein Drp1. The diffusion coefficients measured for the four stable cell lines were compared with predicted ones (Fig. 3A), and the size of the species corresponding to each diffusion coefficient was thereby estimated. The results indicate that the size of Drp1 oligomer present in the cytoplasm of cell lines 24c, 25p and 28p falls in the range of 7–8 nm, which corresponds to a tetramer. For the case of cell line 22p the diffusion coefficient corresponds to a hexamer (Fig. 3A) and even bigger oligomer was detected for the GFP-Drp1 isoform 1. The results indicate that the tetrameric form of Drp1 is the most prevalent in the cytoplasm (Fig. 3A), but a slight concentration dependence suggests that the size of the predominant oligomeric form can increase with the cytoplasmic concentration of Drp1 (Fig. 3B).

**Figure 4:**
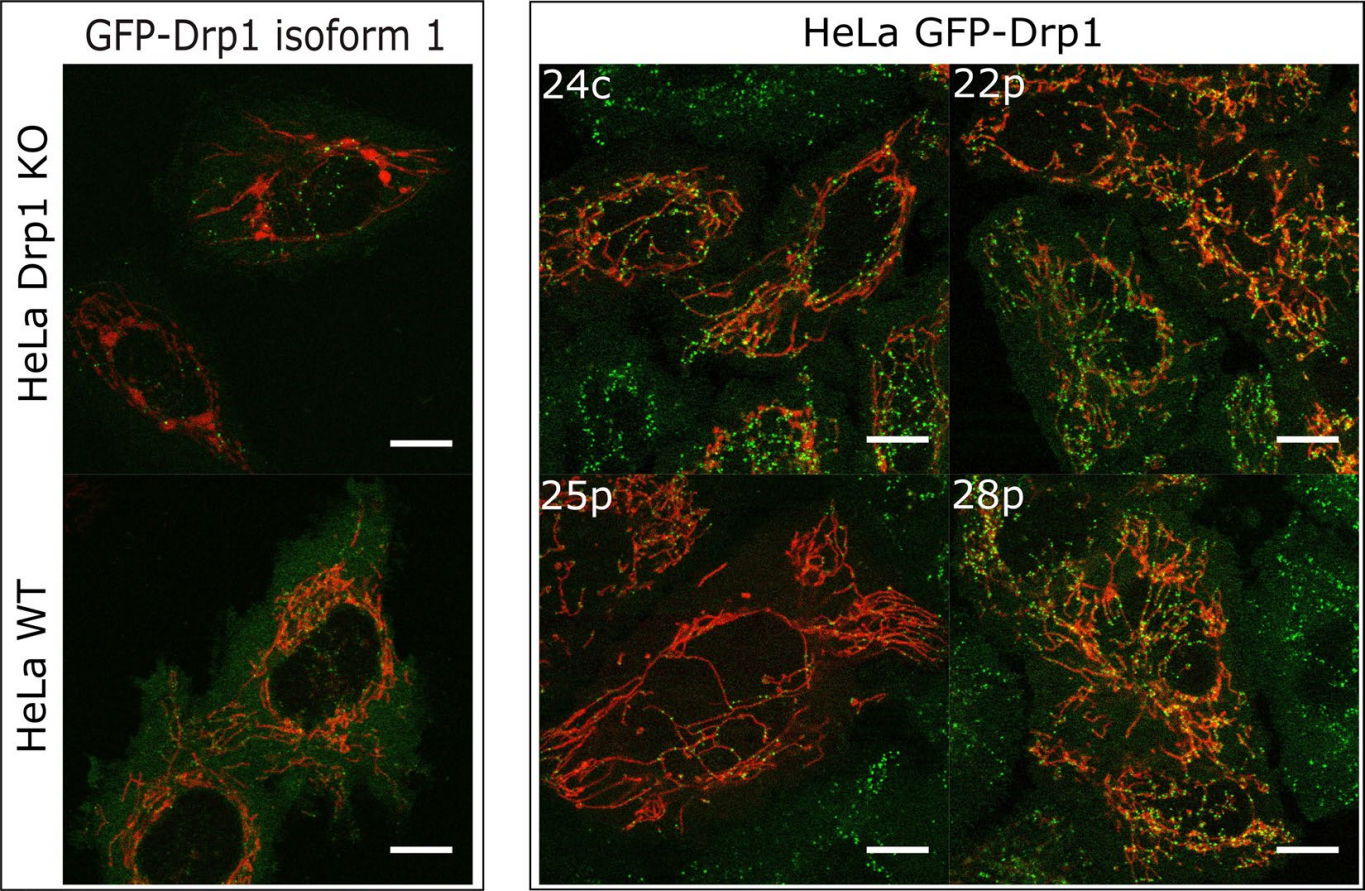
Expression pattern of GFP-Drp1 in transfected HeLa Drp1 KO, in transfected non-modified HeLa (HeLa wt) and in four different gene-edited stable HeLa monoclonal cell lines. Mitochondria are shown in red (transfection with Mito-mNeptune), and the expression of GFP-tagged Drp1 is shown in green. Scale bar, 10 μm.

The expression of GFP-Drp1 in stable and in transfected HeLa cell lines is shown on Fig. 4. For GFP-Drp1 isoform 1 (GFP-Drp1 wt), the protein forms high-intensity puncta corresponding to Drp1 oligomers on the mitochondria. In the stable cell lines, the GFP-Drp1 oligomers on the mitochondria are clearly visible and are much more abundant than in the cells transfected with GFP-Drp1 isoform 1, which shows the advantage of using edited stable cell lines for microscopy studies. The comparison of images obtained for the Drp1 mutants (monomer and dimer) (Fig. 2) with the images of stable cell lines expressing endogenous GFP-Drp1 (Fig. 4) confirms that introduced mutations (K668E, G363D) strongly inhibit the formation of higher order oligomers of Drp1.

**Figure 5:**
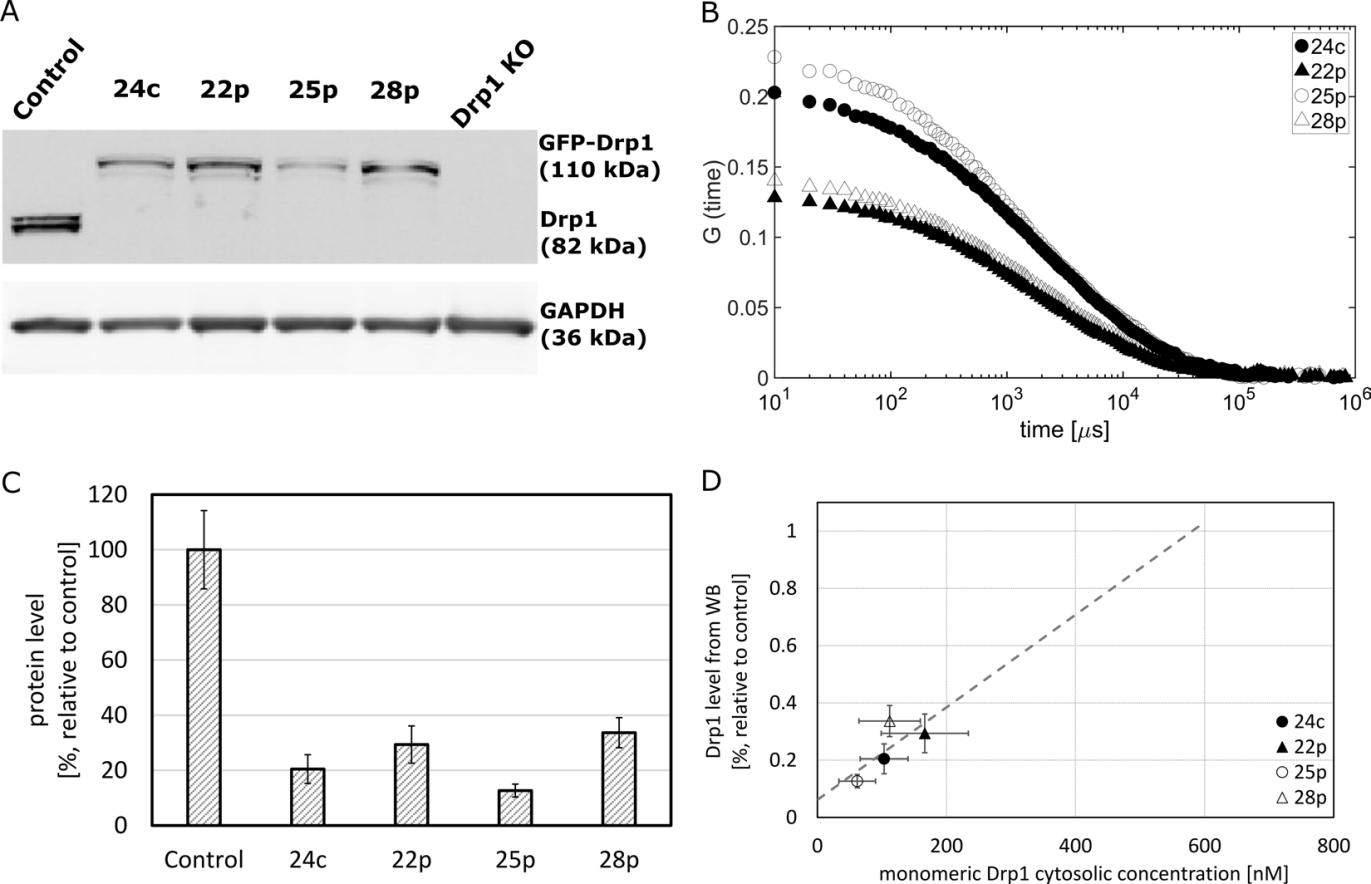
Drp1 concentration in the cell as determined by FCS and WB. (**A**) WB analysis of the levels of Drp1 protein in HeLa wt (Control) and in the gene-edited GFP-Drp1 stable HeLa cell lines as well as in HeLa Drp1 KO. All gene-edited stable cell lines (24c, 22p, 25p, 28p) lack endogenous non-labeled Drp1. Full membrane scans are shown on the Supplementary Fig. S8. (**B**) The autocorrelation curves, G, measured in four different GFP-Drp1 stable cell lines. The values of G at short times (~10 μs) are inversely proportional to concentration and indicating the differences in Drp1 expression levels in the studied cell lines. (**C**) Quantitative analysis of the WB shows differences in the Drp1 expression levels in the HeLa wt and GFP-Drp1 stable HeLa cell lines. (**D**) From the correlation (gray dashed line) of the results obtained by WB and FCS, the concentration of Drp1 in the HeLa wt line can be estimated. FCS results were used to calibrate the WB results. Error bars represent standard deviation.

We showed using western blot (WB) analysis that in the studied stable cell lines, all the Drp1 molecules are tagged with the mEGFP molecule, what is exhibited by a lack of a band of 80 kDa size, which corresponds to untagged Drp1 (Fig. 5A) Thus, there is no background in the form of endogenous untagged protein, and the expression levels are close to the endogenous levels. In the studied cell line of HeLa Kyoto, which has an aneuploidy of approximately 3, we can expect some differences in the GFP-Drp1 expression levels depending on the number of edited alleles. Indeed, in the four studied cell lines, we observed slight differences in expression levels as detected by WB and FCS (Fig. 5B–D). The concentration of tetrameric GFP-Drp1 estimated by FCS in four examined stable cell lines falls in the range 15–42 nM. Since we observed a correlation between GFP-Drp1 level measured by WB and GFP-Drp1 cytosolic concentration measured by FCS (Fig. 5D), we assume that this concentration is higher in the unmodified cell line at approximately 145 nM, which translates to 580 nM when considered as the concentration of monomeric GFP-Drp1 (Fig. 5D).

### Mitochondrial Drp1 exists as a broad distribution of oligomers

**Figure 6:**
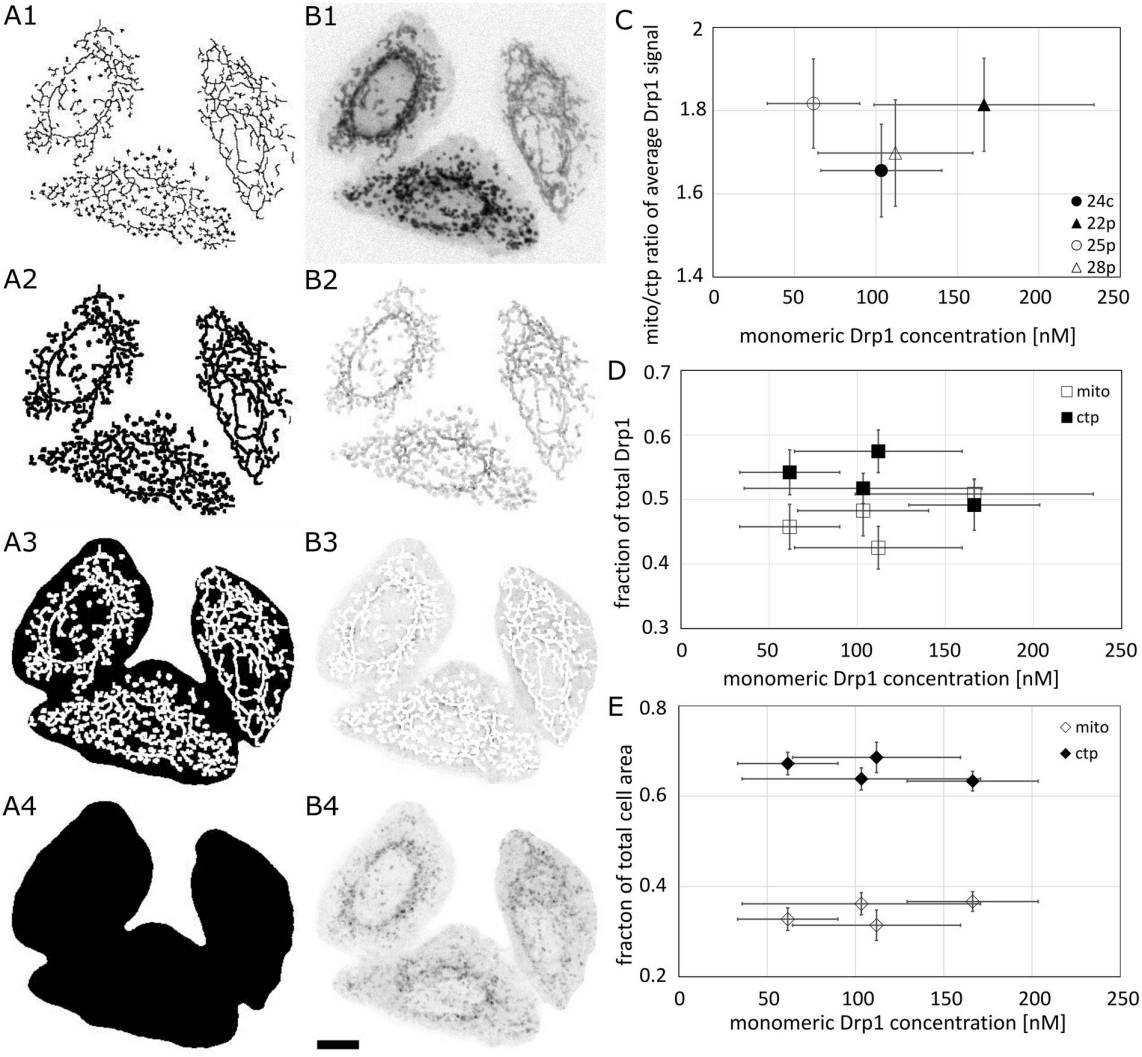
Cytoplasmic and mitochondrial fractions of Drp1 in the cell. The GFP-Drp1 fluorescence signal was measured in different parts of the cell based on the binary masks created for the mitochondria (A2), cytoplasm (A3) and whole cell (A4) based on mito-mNeptune staining (B1). The cytoplasm mask (A3) was created by subtracting the mitochondrial mask from the whole cell mask. The GFP-Drp1 signals corresponding to the mitochondria, cytoplasm and whole cell are shown in B2, B3 and B4, respectively. A1 corresponds to the mitochondrial network detected using the Ridge detection plugin (ImageJ), which was extended by dilation to obtain the mitochondria mask (A2). Scale bar, 10 μm. (**C**) Graph showing correlation between the ratio of mitochondrial to cytoplasmic Drp1 signal in stable HeLa cell lines and monomeric Drp1 concentration. The fraction of total Drp1 (**D**) and fraction of total cell area (**E**) are also shown as functions of the monomeric Drp1 concentration. No clear concentration dependence has been observed for the parameters presented in the figure. Error bars represent standard deviation.

Drp1 is a cytoplasmic protein that can associate with OMMRs. We have analyzed the cytoplasmic and mitochondrial fractions based on confocal fluorescence images (single slice thickness ~ 1 µm). Drp1 is distributed between cytoplasm and mitochondria with approximately 53% of Drp1 in the cytoplasm and the rest of the pool residing on mitochondria (Fig. 6D). Considering that mitochondria occupy less area than the cytoplasm (Fig. 6E), the average concentration of Drp1 at the mitochondria is almost 2-fold higher than that in the cytoplasm (Fig. 6C). We did not observe changes in the fractions of Drp1 in the cytoplasm and on mitochondria, as well as in the ratio of mitochondrial to cytoplasmic GFP-Drp1 fluorescence signal as a function of Drp1 concentration for the concentration range observed in the analyzed cell lines (Fig. 6C,D).

**Figure 7:**
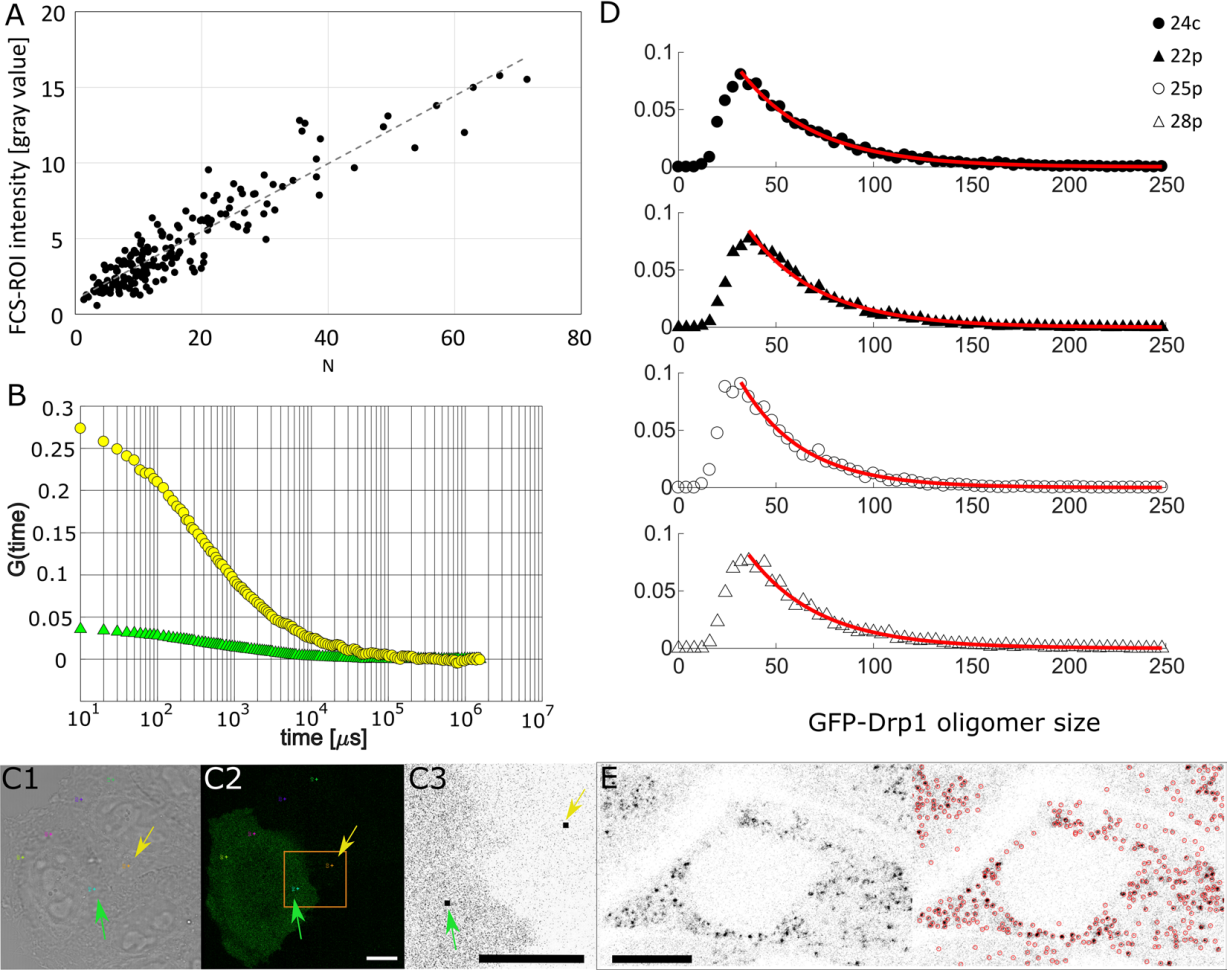
FCS-calibrated imaging. (**A**) Graph presenting correlation between the intensity of the FCS-ROI spot in the confocal image and the FCS-derived number of molecules in the confocal volume (N) in HeLa cells (HeLa Drp1 KO and HeLa wt) transfected with GFP and GFP-Drp1 K668E (black dots). Those parameters were observed to be linearly dependent (gray dashed line), and the linear equation was used to translate the intensities of the Drp1 spots into numbers of Drp1 monomers. (**B**) The different GFP-Drp1 K668E expression levels in HeLa cells enabled the calibration obtained in (**A**). The graph compares the autocorrelation curves obtained in cells with relatively low GFP-Drp1 K668E expression level (yellow circles) and in cells with relatively high GFP-Drp1 K668E expression level (green triangles). The regions corresponding to these FCS curves are marked with a yellow arrow (region of autocorrelation curve presented as yellow circles) and a green arrow (region of autocorrelation curve presented as green triangles) in cells shown in transmitted light (C1) and in the GFP fluorescence channel (C2). (C3) Enlarged region presented in yellow box in C2. The black squares indicated by arrows represent spots whose areas were 8 × 8 pixels, and the mean intensity (gray value) of these areas was used for the calibration presented in (**A**). (**D**) Normalized histograms presenting size distributions of Drp1 spots in stable HeLa cell lines. Sizes of GFP-Drp1 oligomers are presented as the number of Drp1 monomers in detected spots. Red lines represent histograms fitted according to the model of isodesmic supramolecular polymerization, giving the value of the equilibrium constant K_d_. (**E**) Detection of Drp1 spots in TrackMate plugin (ImageJ). The picture taken for analysis is shown on the left, and the red circles on the right represent Drp1 spots detected at a certain threshold. GFP-Drp1 expression is shown in black. Scale bar, 10 μm.

Cytoplasmic Drp1 exists predominantly in tetramer form, according to our translational diffusion measurements (Fig. 3), while the mitochondrial pool of Drp1 forms a dynamic distribution of speckles (Fig. 4, Supplementary Videos 1–4). Recorded movies show the presence of Drp1 speckles of different brightness and sizes which undergo dynamic rearrangements and are able to move along mitochondria. Such observations were already reported^19^ for Drp1, however no detailed analysis of Drp1 oligomers residing at the mitochondria was provided. We applied FCS-calibrated imaging^40^ to estimate the number of GFP-Drp1 molecules forming a particular speckle with a given fluorescence intensity. Using transfected HeLa Drp1 KO and HeLa wt cells with different expression levels of plasmids encoding probes carrying single GFP labels (GFP and GFP-Drp1 K668E), we constructed a calibration curve that translates the local fluorescence intensities in the selected region of interest (ROI) of the image into the number of GFP labels (Fig. 7A,B,C). Using this approach, the fluorescence intensities of the GFP-Drp1 spots were translated into numbers of Drp1 molecules, which allowed us to estimate the average size, ${N}^{{average}}$, of the detected oligomers in the distribution to be 56 ± 4 GFP-Drp1 molecules (average value over four stable cell lines; Table 1).

**Table 1:** Summary of experimentally determined parameters obtained for GFP-Drp1 stable HeLa cell lines, including the cytoplasmic concentration, *c*, expressed in terms of monomeric GFP-Drp1; observed assembly rate, *a*, of GFP-Drp1 prior to fission events preceded by clear Drp1 recruitment; number, *F*, of detected fission events for each cell line; average number, ${N}^{{average}}$, of GFP-Drp1 molecules in the Drp1 oligomers formed at the mitochondria; average number, ${N}_{{fission}}^{{average}}$, of GFP-Drp1 molecules in the active fission complex; number of fission events, *F*_*assembly*_, preceded by clear Drp1 recruitment phase detected in each of the GFP-Drp1 stable cell lines; and obtained value of the fit parameter, *Kc*, of the polymerization model. *Kc* is the product of the equilibrium constant *K* and the cytoplasmic concentration of GFP-Drp1, *c* (see Materials and Methods: equation 4).

cell line name	24c	22p	25p	28p
*c*, [nM]	103 ± 37	166 ± 67	62 ± 28	112 ± 48
*a*, [s^−1^]	3.4 ± 2.9	5.1 ± 3.3	2.9 ± 2.8	2.6 ± 1.7
*F*	55	77	21	33
${N}^{{average}}$	57 ± 4	58 ± 5	50 ± 4	58 ± 5
${N}_{{fission}}^{{average}}$	116 ± 42	100 ± 36	71 ± 22	114 ± 44
*F*_*assembly*_	32	28	14	17
*Kc*	0.90 ± 0.01	0.90 ± 0.01	0.88 ± 0.02	0.89 ± 0.01
*K*_*d*_ = *1/K*, [nM]	29 ± 10	46 ± 19	18 ± 8	31 ± 13

The dissociation constant, *K*_*d*_, corresponds to the polymerization of GFP-Drp1 tetrameric complexes bound to Drp1 OMMRs.

The GFP-Drp1 spots were detected (Fig. 7E) using the TrackMate ImageJ plugin^41^, and each spot intensity was translated into a number of GFP-Drp1 molecules using the calibration curve (Fig. 7A). The resulting distributions of the numbers of GFP-Drp1 molecules in the spots reflect the distribution of GFP-Drp1 oligomers formed in the mitochondrial pool of GFP-Drp1. The observed distributions reveal a broad size range of GFP-Drp1 oligomers reaching more than 100 Drp1 molecules (Fig. 7D). The observed oligomeric size distribution can be interpreted using simple isodesmic supramolecular polymerization model (see Methods, equation 4), from which an estimate of the polymerization dissociation equilibrium constant for the mitochondria-bound form of GFP-Drp1 can be obtained. The K_d_ values obtained are in the 10–50 nM range (Table 1), which shows that relatively high affinity is required to build up large Drp1 oligomers from the Drp1 molecules bound to OMMRs. This oligomerization model suggests that dissociation equilibrium constant for the Drp1 oligomerization is different for the free form of Drp1 residing in the cytoplasm than for the mitochondrial form of Drp1 bound to OMMR. Much stronger interaction occurs between Drp1 bound to OMMR which leads to the formation of Drp1 oligomers composed of more than 100 Drp1 molecules.

### Productive fission complex is composed of around 100 Drp1 molecules and can mature by recruitment of small Drp1 oligomers

**Figure 8:**
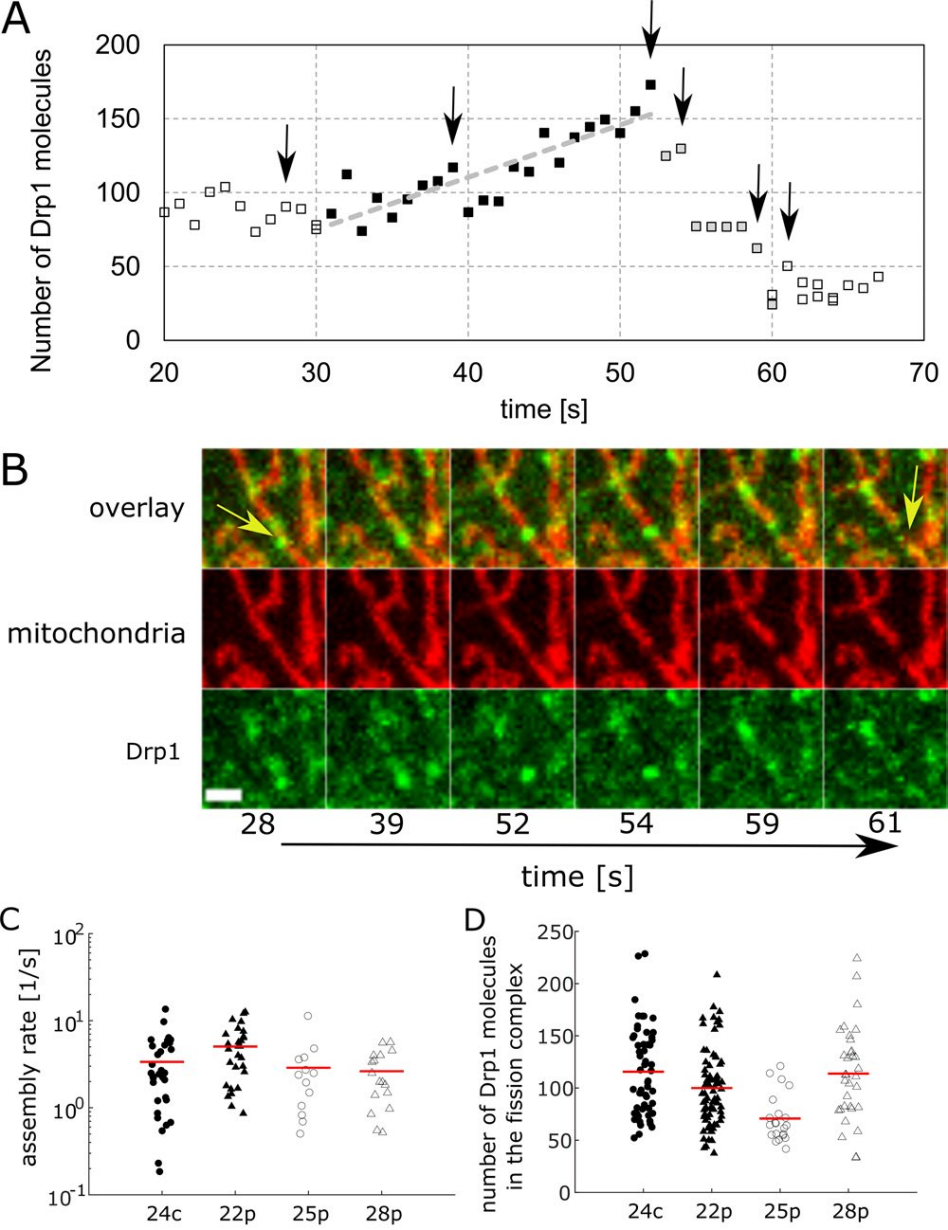
Size of the fission complex. (**A**) Example of mitochondrial fission event presented as changes in the number of Drp1 molecules in the fission complex. Black full squares show the phase of increasing Drp1 oligomer size before the fission event, reaching the maximum size in 54 sec. Gray full squares show the decrease in Drp1 oligomer size after mitochondrial fission. Black arrows mark time points presented in images in (**B**). Gray dashed line is the linear fit of the black squares, which reflects the assembly rate of the Drp1 fission complex before mitochondrial fission. (**B**) Selected frames from the time lapse showing mitochondrial fission event. Yellow arrows indicate the site of the mitochondrial fission event before (28 sec.) and after (61 sec.) the process. Scale bar, 2 μm. (**C**) Plot showing Drp1 oligomer assembly rate before fission event, which corresponds to the slope of the linear fit during the stage when the Drp1 oligomer is increasing in size. Red lines show average values of assembly rate (see Table 1 for exact values). (**D**) FCS-calibrated imaging enabled assessment of the number of Drp1 molecules forming fission complexes in all stable HeLa cell lines. Average sizes of Drp1 fission complexes are shown by red lines. These sizes are 116 ± 42, 100 ± 36, 71 ± 22, and 114 ± 44 Drp1 monomers, and the numbers of analyzed fission events were 55, 77, 21, and 33 for cell lines 24c, 22p, 25p, and 28p, respectively.

We recorded live cell imaging of stable cell lines using a spinning disk microscope and obtained several time-lapse sequences for every cell line, recorded using two channels at every 1 s for a total duration of 5 min. The recorded movies revealed the complex dynamics of Drp1 and the mitochondria (Supplementary Videos 1–4). During careful inspection of obtained time-lapse movies we identified individual fission events which were further analyzed using single particle tracking approach. For every detected fission event a track following a fission complex was obtained and the number of GFP-Drp1 molecules in the fission spots as a function of time was estimated along the trajectory. The average number of GFP-Drp1 molecules detected in the productive fission complex was approximately 100 (Fig. 8D, Table 1, Supplementary Video 5). For around half of the detected trajectories containing fission events (91 out of 186 detected trajectories during which fission occurred), we observed a characteristic pattern of assembly (Fig. 8A, Supplementary Video 5) and disassembly before and after the fission event. Before the actual fission, a maturation phase occurred in which molecules were recruited at a rate of 3 ± 1 GFP-Drp1 molecules/s, (Fig. 8C, Table 1) until the spot reached its maximum value, after which the fission process began, resulting in mitochondrial fragmentation. For some of the fission events no clear recruitment phase was observed (Supplementary Video 6, Supplementary Fig. S2).

The histograms of the sizes of GFP-Drp1 mitochondrial complexes (Fig. 7D) give an estimate that not more than 14% of all detected complexes are larger than 100 GFP-Drp1 molecules, which could explain the relatively low number of fission events observed during live cell imaging. Indeed, the number of identified fission events (Table 1) in the total 105 min of recorded movies (several movies of 5 min duration each, with time steps of 1 second) gives an estimated value of approximately 2 fission events per minute in a square field of view of size of 108 μm × 108 μm. Approximately 10 cells on average appeared in each such area, giving approximately 0.2 fission events per minute per cell. In other words, for a single cell in the time lapse movie, we must wait on average approximately 5 min to observe a single fission event. Notably, identifying fission events by eye is a cumbersome activity, indicating the need to develop an efficient automated procedure for the analysis of such complex time-lapse data.

### Niclosamide treatment induces increased oligomerization of Drp1

**Figure 9:**
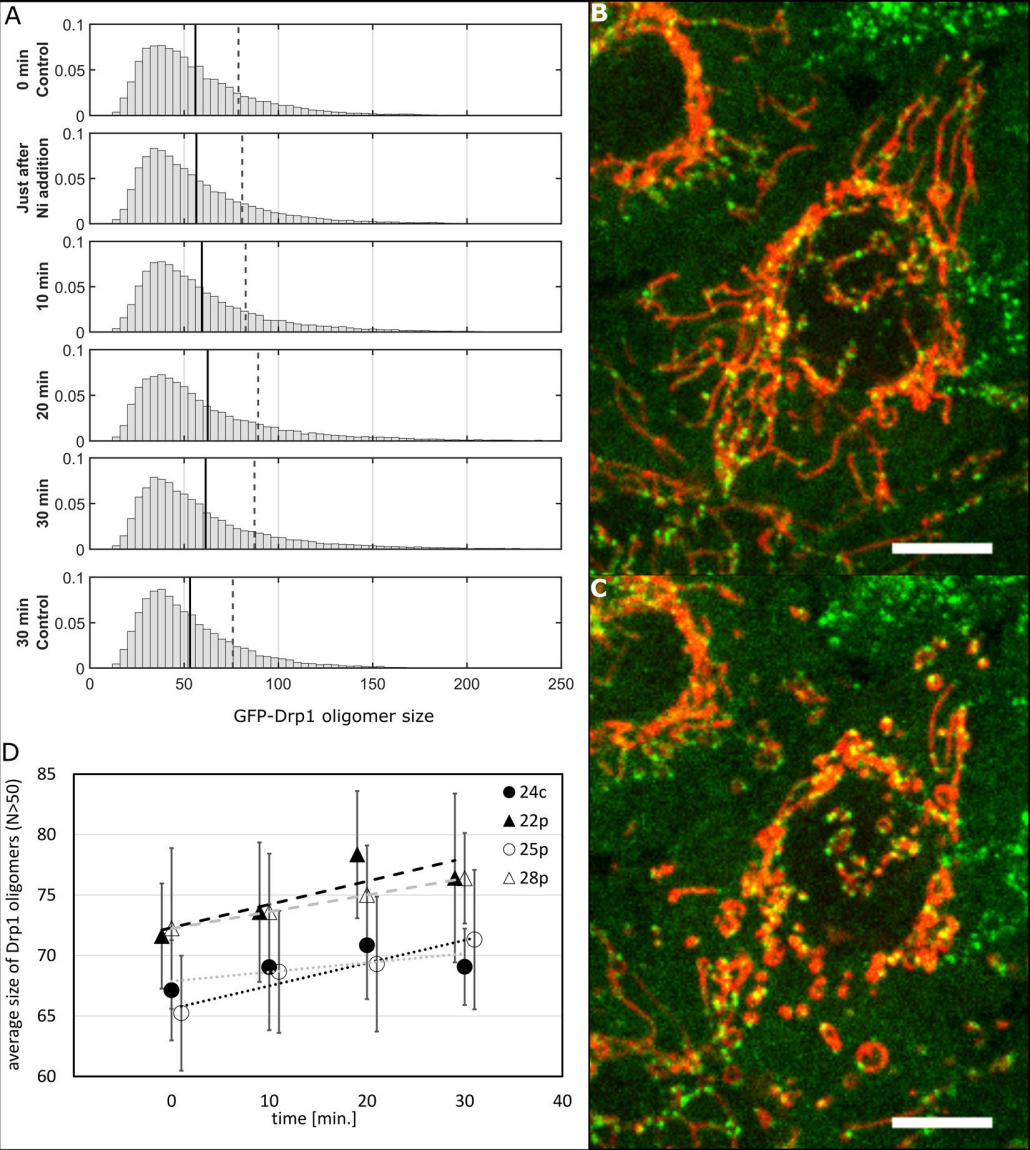
Niclosamide addition to stable HeLa cells increases the average Drp1 oligomer size. (**A**) Comparison of normalized histograms for stable HeLa cell line 22p at different time points during niclosamide treatment. Black lines represent average sizes of all detected Drp1 oligomers, and the gray dashed line shows the average sizes of Drp1 oligomers larger than 50 Drp1 monomers. Representative image of mitochondrial network before niclosamide addition (**B**) and 10 min after niclosamide addition (**C**) in stable HeLa cell line 22p. Scale bar, 10 μm. (**D**) The average sizes of Drp1 oligomers larger than 50 Drp1 monomers increase over time after niclosamide treatment. Dashed and dotted lines present linear fit reflecting the increasing tendency in Drp1 oligomer size over time after niclosamide treatment for every edited cell line. Error bars represent standard error of the mean. Mitochondria are shown in red (transfection with Mito-mNeptune), and the expression of GFP-tagged constructs is shown in green.

Comparison of the distribution of oligomers in the mitochondrial pool of Drp1 and the number of Drp1 molecules in the productive fission complex indicates that only a fraction of Drp1 oligomers reach the size threshold, N_fission_, becoming large enough to perform fission. Only a subset of the Drp1 oligomers that surpass the N_fission_ threshold actually perform fission (Supplementary Videos 7, 8, Supplementary Figs S3, S4). This result suggests that increased fission can be achieved by increasing the number of Drp1 oligomers that surpass the N_fission_ threshold. We applied niclosamide treatment to the cells and followed the GFP-Drp1 distribution over time. Niclosamide is known to trigger mitochondrial fragmentation in a Drp1-dependent manner^42^. Indeed, we have observed a clear fragmentation (Fig. 9B,C) upon treatment of HeLa cells with 10 µM niclosamide. We observed an increase in the number of larger GFP-Drp1 oligomers upon niclosamide treatment (Fig. 9A,D) which confirm our predictions.

## Discussion

In this work, we provide a quantitative description of the distribution of the GFP-Drp1 in the HeLa cell line, in order to estimate oligomeric states of this protein in the cytoplasm and on mitochondria. By measuring the diffusion coefficients of GFP-Drp1 and interpreting the results using LDVM, we determined that the predominant form of this protein in the cytoplasm of HeLa cells stably expressing GFP tagged endogenous Drp1 is a tetramer, which forms a reservoir of Drp1 available for recruitment to mitochondria. This is consistent with other reported experiments, performed using FRAP, which showed that Drp1 wt (isoform 3) diffused slower than Drp1 G363D mutant, what indicated that cytosolic Drp1 is tetrameric or larger^30^. Other reports suggested that Drp1 can form reservoirs in cytosol (with the size of around 50 nm), from which fission competent Drp1 dimers can dissociate to form fission complex around mitochondria^27^. However, our diffusion results show that the predominant form of GFP-Drp1 in the cytoplasm of stable HeLa cell lines is a tetramer, but we cannot exclude the presence of very small number of larger Drp1 oligomers in the cytoplasm (Supplementary Methods 1). We observed some concentration dependence of the measured diffusion coefficients (Fig. 3B), indicating that the size of the oligomeric form in the cytoplasm might increase with the concentration of GFP-Drp1. This observation could mean that the basic Drp1 unit recruited to the mitochondria is tetrameric at Drp1 concentrations of approximately 100 nM, while higher oligomeric forms might be formed at higher concentrations (Fig. 3). This observations is in agreement with experiments performed *in vitro* by Macdonald and colleagues, who showed that the Drp1 (isoform 3) elution profile in size exclusion chromatography changed as a function of loaded protein concentration^27^. In addition, the size of the oligomers of the GFP-labeled isoform 1 of Drp1 observed in cytoplasm of HeLa cells was greater than the size of the Drp1 oligomers observed for an endogenous combination of Drp1 isoforms in cytoplasm of stable HeLa cell lines obtained by gene editing (Fig. 3). In comparison to transfected HeLa cells, in which only isoform1 of GFP-Drp1 is expressed, stable HeLa cell lines studied in the present work can express a mixture of GFP tagged Drp1 isoforms, using exon 1 which was modified to contain a GFP coding sequence. It was reported that the most abundant isoforms of Drp1 in HeLa cells are isoforms 1, 2, 3 and 4^43^ (see Supplementary Table S2 for description of Drp1 isoforms). This result shows the importance of combining of Drp1 isoforms for the correct assembly of Drp1 rings. Indeed, different Drp1 isoforms were reported to have different oligomerization properties^33^. We observed a clear difference in the fluorescence pattern of GFP-Drp1 in the stable cell lines and in the transfected HeLa cells (Fig. 4). The number of GFP-Drp1 oligomers (green dots) residing on the mitochondria is much larger in the stable cell lines than in the transfected cells. Using the CRISPR/Cas9 gene-editing technique, we were able to measure GFP-tagged endogenous Drp1 in HeLa cells, which enabled observations under conditions very close to the native ones. In all four stable HeLa cell lines examined, the whole Drp1 population was tagged with GFP, eliminating the problem of background of the non-labeled endogenous Drp1, which could interfere with the obtained results. The concentrations of the cytoplasmic pool of tetrameric GFP-Drp1 in the stable cell lines were in the range 15–42 nM (60–168 nM for monomeric GFP-Drp1 species). The comparison of Drp1 levels between the gene-edited cell lines and the HeLa wt (unmodified cell lines) indicates that the concentration of Drp1 is higher in the unmodified cell line (Fig. 5) amounts to approximately 0.58 μm (in terms of the monomeric species). This value is similar to Drp1 concentration measured by quantitative Western blotting in U2OS cell line, which was around 0.5 μM^24^. We estimated that 40–50% of the cellular pool of GFP-Drp1 localizes to the mitochondria, while the rest remains in the cytoplasm (Fig. 6). Such estimates differ significantly from other report, in which only approximately 3% of the total amount of Drp1 was reported to cofractionate with mitochondria^44^, however more recent studies report 49% of total Drp1 puncta were associated with mitochondria^19^.

The mitochondrial pool of GFP-Drp1 was analyzed using FCS-calibrated imaging to translate the intensities of individual GFP-Drp1 spots detected on the mitochondria into the numbers of GFP-Drp1 molecules forming the GFP-Drp1 complex (seen as a fluorescence spot). The distributions of the numbers of GFP-Drp1 molecules in the spots formed by the mitochondrial pool of Drp1 indicate a broad size range of the GFP-Drp1 complexes. The characteristic equilibrium coefficient describes the distribution of oligomers and corresponds to the interaction between GFP-Drp1 bound to OMM Drp1 receptors. In agreement with other studies^19^ we observed increased oligomerization at the mitochondria, which indicates that interactions with mitochondrial components (e.g., OMM Drp1 receptors or cardiolipin) are necessary for the higher order oligomers formation. The estimated value of the observed equilibrium dissociation constant for oligomerization was 31 nM (average value over four stable cell lines (Table 1)). These results complement the results obtained by Wei-ke Ji and colleagues^19^, who proposed that Drp1 exists in dynamic equilibrium on mitochondria, since they observed the presence of Drp1 oligomers of different sizes residing on mitochondria which were able to move along this organelle. Same authors observed that not all Drp1 complexes associated with mitochondria lead to their fission, however they reported a coincidence of mitochondrial fission with high-threshold Drp1 puncta. Nevertheless, the distribution of sizes of Drp1 oligomers has not been described previously and is provided in the present work. From our analysis we conclude that the average number of GFP-Drp1 molecules forming the fission complex that actually performs fission is around 100. This shows that only a fraction of Drp1 oligomers are capable of mitochondrial fragmentation, explaining the relatively small number of fission events observed using live cell imaging. Based on this point and our observations concerning size of the fission complex, we propose two possible mechanisms for the upregulation of the mitochondrial fission in the cell.

In the first mechanism, the increase in the number of fission signals leads to the activation of sufficiently large but inactive Drp1 rings, resulting in the increase in the fission rate. In the second mechanism, the change in the GFP-Drp1 distribution increases the fraction of sufficiently large Drp1 rings, which can be triggered by existing fission signals leading to increased fission. We have treated stable cell lines expressing GFP-Drp1 with niclosamide (Fig. 9), which induces mitochondrial fission in a Drp1-dependent manner^42^. Niclosamide treatment induced a clear change in the structure of the mitochondrial network in the studied cell lines (Fig. 9B,C). We show that the addition of this reagent to GFP-Drp1 stable HeLa cell lines increased the average size of the Drp1 oligomer, indicating that upregulated fission was accompanied by an increase in the average GFP-Drp1 oligomer size, what supports the second proposed mechanism of fission regulation (Supplementary Fig. S5).

For approximately 49% of detected fission complex trajectories, we observed the characteristic assembly process preceding an actual fission event. The assembly progressed at the average rate of 3 to 5 GFP-Drp1 molecules per second (Table 1, Fig. 8C), which could correspond to the attachment of one GFP-Drp1 tetramer every second and could result in the growth of a selected oligomer by 40 molecules within 10 seconds. This result suggests that in addition to the proposed mechanism of fission complex maturation, where two larger complexes can merge to form an even larger complex^19^, another mechanism can be considered in which smaller tetrameric GFP-Drp1 complexes can be attached step by step to the growing complex until it reaches the point where fission is executed.

Analysis of the trajectories containing individual fission events indicates that there is no very sharp threshold for the size of the complex after which fission is performed, since we observed complexes that grew to 200 GFP-Drp1 molecules before the completion of fission. The observed number of Drp1 molecules forming a fission complex could be explained by several structural models of Drp1 ring, however the exact organization of the Drp1 molecules in the fission complex cannot be obtained from our data because of insufficient spatial resolution. Other researchers reported the diameter of the fission complex of 130–150 nm^22,23^ corresponding to 48 Drp1 tetramers forming mitochondrial constriction site^22^. Other reports suggest sizes of Drp1 fission rings in a smaller range around 30–40 nm which are composed of rings formed by 16–20 Drp1 molecules^20,21^. If several such rings form at the fission site, they can form a bigger Drp1 oligomer, which based on our results is composed of around 100 molecules. The estimates based on the superresolution microscopy give support to such model in which fission ring extends over the length of 68 nm corresponding up to 4 helical rings formed around mitochondria^23^. Our results complement the existing literature data concerning a mechanism of mitochondrial fission in which Drp1 plays a crucial role. The parameters collected from various studies which describe the distribution and interactions of Drp1 in the cell could serve as a starting point for the development of a robust chemical biology model describing the regulation of mitochondrial fission by Drp1.

## Methods

### Plasmids and gene editing

The cDNA encoding isoform 1 (transcript variant 1, NP_036192, 736 aa, Supplementary Table S2) of Drp1 was purchased from OriGene (RG221708). The cDNA was amplified by PCR using forward (for) and reverse (rev) primers (for: AGC TTC GAA TTC TAT GGA GGC GCT AAT TCC TGT CAT AAA CAA GCT C, rev: CTG AAA TCC GGG AGA CTC ATC TTT GGT GAG GAT CCA CCG GA) and subcloned into the pmEGFP-C1 vector using the restriction enzymes EcoRI and BamHI. The pmEGFP-C1 vector was a gift from Benjamin Glick (Addgene plasmid #36412). The K668E and G363D mutants of Drp1 were obtained by QuikChange site-directed mutagenesis^45^ and confirmed by DNA sequencing. The primers used for obtaining Drp1 mutants were as follows: K668E, for: GTG CCA GAG GCA GTA ATG CAT TTT TTG GTT, rev: TAC TGC CTC TGG CAC ACT GTC TTG AAT ATT; G363D, for: TGC GGT GAT GCT AGA ATT TGT TAT ATT TTC CAT GAG, rev: TCT AGC ATC ACC GCA TAG CTC CGA A. The plasmids were amplified using Endotoxin-Free Maxiprep Kits (Sigma-Aldrich). The mito-mNeptune plasmid was obtained by replacing the DsRed tag sequence in pDsRed2-Mito from Clontech with the mNeptune sequence from mNeptune2-C1 (mNeptune2-C1 was a gift from Michael Davidson (Addgene plasmid #54836)) using the restriction enzymes BamHI and NotI. The sequence encoding mNeptune was amplified using the following primers: for: GTT GGG GGA TCC ACC GGT CGC CAC CAT GGT GTC TAA GGG CGA AGA GC, rev: AGA GTC GCG GCC GCT ACT TGT ACA GCT CGT CCA TGC CAT TAA G. The GFP-ferritin plasmid was obtained using a plasmid encoding ferritin heavy chain 1 (FTH1, OriGene) and pmEGFP-C1. The sequence encoding FTH1 was subcloned into the pmEGFP-C1 vector using the restriction enzymes BglII and EcoRI and the following primers for FTH1 amplification: for: GGA CTC AGA TCT TCC GGC GCA GCA GCA GGT GGA GGT TCG GGT GGA GGT AGC GGT GGA GGT ATG ACG ACC GCG TCC ACC, rev: ACT GCA GAA TTC TTA GCT TTC ATT ATC ACT GTC TCC CAG GGT G.

Gene editing was performed using plasmids U6gRNA-Cas9-2A-RFP encoding the guiding RNA together with the Cas9 enzyme (Sigma-Aldrich) in a single plasmid. We selected two potential guiding RNAs, both of which showed cleavage activity. The PAM sequences were located before the first exon (Custom sequence) or in the first exon (Predesigned sequence - HS0000233669) of the Drp1 gene. Cells were seeded into 6-well plates and transfected as described. Single-plasmid transfection (Custom or Predesigned) was used to obtain Drp1 KO cell lines. Cotransfection with a plasmid coding GFP surrounded by homology arms was used to obtain cell lines with GFP is incorporated into their genome and fused to Drp1 protein at its N-terminus. Twenty-four hours after transfection, the cells were taken for FACS sorting to select the population of cells with high Cas9 enzyme content as reported by the presence of RFP. The selected cells were single-cell plated in 96-well tissue culture plates and left for approximately 2 weeks. Then, single colonies were identified and checked for the presence of Drp1 by WB (KO, GFP-tagging) or for the size of the PCR product surrounding the editing site (PAM) by PCR based on genomic DNA extracted from the obtained cell lines (Supplementary Figs S6, S7).

### Genomic DNA extraction and PCR

The genomic DNA of edited HeLa Kyoto cells was extracted using QuickExtract DNA Extraction Solution (Epicentre) according to the manufacturer’s protocol. To check GFP incorporation upstream of the first exon of DNM1L in the genome of HeLa Kyoto cells, we used PCR primers complementary to sites outside the homology arms. The sequences of those primers were as follows: for: CAA AAC CCC TGG AGA ACC CG, rev: TCG CAG ACC AAG GAA ATG TGT.

### Cell culture and transfections

HeLa Kyoto cells were maintained in Dulbecco’s modified Eagle medium (DMEM) containing 1 g/L glucose and supplemented with 10% fetal bovine serum (FBS, Gibco), 2 mM L-Glutamine (Gibco) and 1% penicillin/streptomycin (Sigma-Aldrich). For transfection, cells were seeded into appropriate dishes approximately 24 hours before the procedure. Transfection was performed with the jetPRIME Transfection Reagent (Polyplus Transfection) using 1 μL of reagent for 500 ng of plasmid DNA. Four hours after transfection, the medium was changed. Approximately 24 hours after transfection, cells were taken for microscopic observation.

### Western blotting

Cells growing in 6-well plates were treated with trypsin (Sigma-Aldrich), washed with PBS and sedimented by centrifugation. Next, the cell pellets were lysed using RIPA lysis buffer supplemented with protease inhibitors (Sigma-Aldrich) and incubated on ice for 30 min. The lysates were centrifuged at 17000 × *g* for 30 min at 4 °C, and the supernatant was collected. The protein concentration was measured using the Bradford method. Lysates were mixed with Bolt LDS Sample Buffer (Life Technologies) supplemented with β-mercaptoethanol (final concentration 5%). Samples containing equal amounts of protein (20 μg) were separated by SDS-PAGE on gradient gels (4–12%, from Invitrogen) and transferred to nitrocellulose membranes (Bio-Rad Laboratories). After blocking in Odyssey Blocking Buffer diluted 1:1 in TBS for 1 hour, the membranes were incubated with primary antibodies at 4 °C overnight. After washing with TBS-T, the blots were incubated with secondary antibodies for 1 hour at room temperature. The blots were visualized using the Odyssey Infrared Imaging System (Li-Cor Biosciences, Lincoln, NE, USA). Drp1 was detected using mouse antibody (BD Bioscience) at a 1:2000 dilution. Rabbit anti-GAPDH (Abcam) was used at 1:50000 dilution. Fluorescently labeled secondary antibodies (Li-Cor Biosciences) were used at 1:20000 dilution.

### FCS measurements and analysis

FCS measurements were performed on a Leica SP8 commercial setup equipped with PicoQuant electronics for time-correlated single-photon counting (TCSPC) detection and single-photon avalanche diode (SPAD) detectors for single-molecule detection. Light was focused using a water immersion objective with a numerical aperture of 1.2. During FCS measurements the confocal spot was positioned in the cytoplasm of HeLa cells in order to obtain diffusion coefficients for GFP-Drp1 residing in cytoplasm. Measurements were performed and according to established protocols^46–49^ and further details on FCS acquisition and analysis are given in the Supplementary Methods 1.

### FCS-calibrated live cell imaging

FCS curves were measured for molecular species containing a single GFP fluorophore, including GFP and GFP-Drp1 K668E (monomer), to obtain the relation between the number of molecules measured in the FCS experiment (Fig. 7B) and the intensity of the image in the FCS measurement spot acquired with a photomultiplier tube (PMT) detector (Fig. 8C3). The same laser power was used for all measurements to secure a constant molecular brightness of GFP. Images were acquired with the PMT detector prior to FCS measurements in the 1024 × 1024 format with a pixel size of 64 nm. The intensity of the signal, ${I}_{{FCS}-{ROI}}$, from the 8 × 8(~500 nm) pixel ROI surrounding the FCS spot was related to the number of molecules (N) measured by FCS (Fig. 7A). The calibration was obtained as a linear fit,

2 $${I}_{FCS-ROI}(N)=a\times N+b.$$

With *a* = 0.224 ± 0.014 and *b* = 0.98 ± 0.28. The measurements were performed using a Leica SP8 confocal laser scanning microscope (LSM). Using this calibration (equation 2), we could estimate the number of GFP-Drp1 molecules in each spot and quantitatively interpret the obtained distribution of intensities of GFP-Drp1 spots observed in stable GFP-Drp1 HeLa cell lines (Fig. 7D).

### Live cell imaging, confocal microscopy and niclosamide treatment

Cells were visualized using the 63 × water objective at 37 °C on the LSM SP8 (Leica) and on a spinning disk microscope (Zeiss). For the analysis of mitochondrial fission events, GFP-Drp1 stable HeLa cells were seeded at 1 × 10^4^ cells in 4-well LabTek dishes two days before imaging. Twenty-four hours before imaging, these cells were transfected as described with the mito-mNeptune plasmid. The cells were then imaged using a spinning disk microscope in live imaging medium (LIM, Molecular Probes). Time-lapse images were collected every 1 second for 5 min in a single focal plane. For FCS measurements on the LSM SP8, HeLa cells were likewise seeded in 4-well LabTek dishes at 1 × 10^4^ cells per well. For niclosamide (Sigma-Aldrich) treatment, GFP-Drp1 HeLa Kyoto cells were seeded at 5 × 10^3^ cells in 8-well LabTek dishes two days before imaging. Live cells were visualized in LIM under an LSM SP8 confocal microscope. Four or five Zstacks were taken under every condition (before niclosamide addition; just after addition; 10, 20 and 30 min after addition; 30 min after addition in control cells (without niclosamide addition) and in cells treated only with DMSO - data not shown). Niclosamide was used at a final concentration of 10 μM. Each Zstack step was 0.8 μm.

### Analysis of cytoplasmic and mitochondrial fractions

The images of GFP-Drp1 stable HeLa cell lines (24c, 22p, 25p, 28p) transfected with mito-mNeptune for staining of the mitochondria were acquired using a Zeiss spinning disk microscope. For every cell line five fields of view were analyzed, what gave in total about 20 cells per cell line. The images obtained were analyzed using ImageJ to estimate the cytoplasmic and mitochondrial fractions of Drp1. The mitochondrial fraction of Drp1 was taken as the GFP-Drp1 signal limited to the area covered by the mitochondrial mask (Fig. 7A2). The mitochondrial mask was generated by single dilation of the binary image of the mitochondrial network (Fig. 7A1) obtained using the ImageJ Ridge detection plugin. The mask of the whole cell (Fig. 7A4) was generated based on the mito-mNeptune signal (Fig. 7B1) using ImageJ (“MinError” thresholding” combined with dilation and median filtering operations). The mask for the generation of the cytoplasmic Drp1 fraction was generated by subtracting the mitochondrial mask from the whole cell mask.

### Analysis of the distribution of mitochondrial Drp1 and histogram matching

The distribution of GFP-Drp1 intensities was obtained by using the TrackMate plugin to detect spots corresponding to GFP-Drp1 oligomers (red circles on Fig. 8E). A certain threshold for the detection of spots was used to avoid the detection of noise (i.e., spots outside the cells). The smaller oligomers are underestimated in the resulting histograms because of the insufficient signal-to-noise ratio for dimmer objects. The spot intensities obtained were translated into a distribution of GFP-Drp1 oligomers by calculating the number of GFP-Drp1 molecules in each spot using the calibration curve, ${I}_{{FCS}-{ROI}}(N)$ (equation 2), obtained from FCS-calibrated imaging. We analyzed the distributions of GFP-Drp1 oligomers using a simple oligomerization model^50,51^ and obtained the equilibrium constant for the polymerization of GFP-Drp1 on mitochondria. In this model, the oligomerization process is described by a simple set of reactions in which a larger oligomer (*i*-mer, at concentration *X*_*i*_) is formed by the attachment of basic components (e.g., tetramer, at concentration *X*_*1*_) to the existing oligomer (*(i − 1)*-mer, at concentration *X*_*i−1*_), with an equilibrium constant, *K*, which is independent of the oligomer size:

3 $${X}_{1}+{X}_{i-1}\mathop{\to }\limits^{K}{X}_{i}$$

This set of reactions leads to a distribution of sizes (equation 4) that can describe our experimental data (Fig. 7D, red lines):

4 $$f(n)={A}_{1}{(Kc)}^{n-1}$$

In the above formula, *n* is the degree of oligomerization, *K* is the equilibrium constant for attaching another building block to an existing oligomer, *c* is the concentration of the smallest building block (a monomer, a dimer, or a tetramer as in our case), and *A*_*1*_ is a constant that depends on several parameters including *c* and cell volume. The value of *Kc* was obtained for each GFP-Drp1 stable cell line by fitting of the model to the experimental distribution of GFP-Drp1 oligomers (Table 1). The corresponding equilibrium constant, *K*, was calculated from the obtained fit parameter *Kc* divided by the cytoplasmic concentration of GFP-Drp1 (Table 1). The dissociation constant, *K*_*d*_, was calculated as the inverse of the equilibrium constant, *K*_*d*_* = 1/K*. The translation of the spot intensity to the number of GFP-Drp1 molecules was also performed for time-series images obtained using spinning disk microscopy. The calibration was transferred between microscopes using histogram matching between images obtained in the spinning disk and LSM confocal microscopes (Supplementary Methods 2).

### Calculation of the diffusion coefficients and hydrodynamic radius from structural data and LDVM

The size of any given macromolecule was determined as a hydrodynamic radius, *r*_*p*_, defined through the Einstein-Stokes relation (equation 1). Based on the available structural data (Protein Data Bank (PDB) 4BEJ and electron microscopy (EM)^22^) the diffusion coefficients in water, *D*_0_, were calculated for molecules of GFP, ferritin, Drp1 monomer, Drp1 dimer and different Drp1 oligomers using HydroPro software^52^. The calculations were performed for a temperature of 37 °C and the corresponding water viscosity *η* = 0.69 mPa using structures presented in Supplementary Fig. S1. The resulting diffusion coefficients were used to estimate the size of the diffusing molecules, given as the hydrodynamic radius *r*_*p*_, calculated using the Einstein-Stokes relation (equation 1). The hydrodynamic radii *r*_*p*_ for the test probes and Drp1 oligomers from Supplementary Fig. S1 are summarized in Supplementary Table S1.

LDVM depends on four parameters that describe the properties of the diffusing object and the characteristic length scales of the surrounding medium (*R*_*h*_, $\xi$) and on two constants (*a*, *A*), the latter giving the estimate for the viscosity of the cytoplasm (*A* × *η*_0_) for probes smaller than $\xi$ (equation 5).

5 $$ln(\frac{\eta ({r}_{p})}{{\eta }_{0}})=ln(A)+{(\frac{{\xi }^{2}}{{R}_{h}^{2}}+\frac{{\xi }^{2}}{{r}_{p}^{2}})}^{-a/2}$$

The parameters of the LDVM were taken from the literature (*A*=1.3 ± 0.3, $\xi$ = 5 ± 4 nm, *R*_*h*_ $\approx$ 8  nm, and *a* = 0.49 ± 0.22)^53^ except for the parameter *A*, which was allowed to change to best fit the experimental data obtained for our calibration probes (GFP, Drp1 monomer and dimer, GFP-ferritin). Based on the obtained value of *A* = 1.5, the estimated viscosity of the cytoplasm at nanometer length scales in the studied HeLa Kyoto cell line was (*A* × *η*_0_) ~1 mPa. The diffusion coefficients in the cytoplasm of the HeLa cells were calculated using the Einstein-Stokes relation (equation 1), with the viscosity *η*(*r*_*p*_) calculated for each probe of size *r*_*p*_ using equation 5. The predicted diffusion coefficients as a function of the size of the probe *r*_*p*_ are shown in Fig. 1.

### Image analysis – detection of GFP-Drp1 spots

To analyze the number of Drp1 molecules in spots in the cell, the ImageJ plugin TrackMate (v3.5.1) was used to detect these spots at a certain threshold based on confocal microscopy images obtained using the LSM SP8. The parameters used in TrackMate were as follows: estimated blob diameter −512 nm; LoG detector settings: tracker – Simple LAP tracker, linking max distance and gap-closing max distance −750 nm, gap-closing max frame gap −0. The fluorescence intensities of the detected spots were translated into numbers of Drp1 molecules using the linear equation obtained from FCS-calibrated live cell imaging (equation 2).

### Detection and analysis of fission events

Individual mitochondrial fission events selected for analysis were detected by eye based on time-lapse images obtained using a spinning disk microscope. For the analysis of mitochondrial fission events, TrackMate was used ref.^22^ to obtain individual tracks of GFP-Drp1 spots. The following TrackMate settings were used: estimated blob diameter −512 nm; LoG detector settings: tracker −LAP tracker, frame-to-frame linking max distance and track segment gap-closing max distance −750 nm, gap-closing max frame gap −2, track segment splitting −750 nm and track segment merging – 500 nm. The intensities of the spots in the detected tracks were translated into numbers of Drp1 molecules using *I*_*SD*_ calibration (Supplementary Methods 2, equation S11).

### Data availability

The datasets generated during and/or analyzed during the current study are available from the corresponding author on reasonable request.

## Electronic supplementary material

Supplementary Video 1

Supplementary Video 2

Supplementary Video 3

Supplementary Video 4

Supplementary Video 5

Supplementary Video 6

Supplementary Video 7

Supplementary Video 8

Supplementary_Information_file
