# Supplementary material for: Insight into the fission mechanism by quantitative characterization of Drp1 protein distribution in the living cell
Source: Sci Rep. 2018 May 25;8:8122. doi: 10.1038/s41598-018-26578-z (PMC5970238; doi:10.1038/s41598-018-26578-z)
Supplement: Supplementary file 9 — Supplementary_Information_file [file 41598_2018_26578_MOESM9_ESM.pdf]

Supplementary information

Insight into the fission mechanism by quantitative characterization of Drp1 protein distribution in the living cell

Bernadeta Maria Michalska[1], Karina Kwapiszewska[2], Joanna Szczepanowska[1], Tomasz Kalwarczyk[2], Paulina Patalas-Krawczyk[1], Krzysztof Szczepański[2], Robert Hołyst[2], Jerzy Duszyński[1], Jędrzej Szymański[1]*

[1] Laboratory of Bioenergetics and Biomembranes, Department of Biochemistry, Nencki Institute of Experimental Biology of Polish Academy of Sciences, 3 Pasteur Str., 02-093 Warsaw, Poland

[2] Institute of Physical Chemistry of the Polish Academy of Sciences, Kasprzaka 44/52, 01-224 Warsaw, Poland

* Corresponding author: Jędrzej Szymański, j.szymanski@nencki.gov.pl

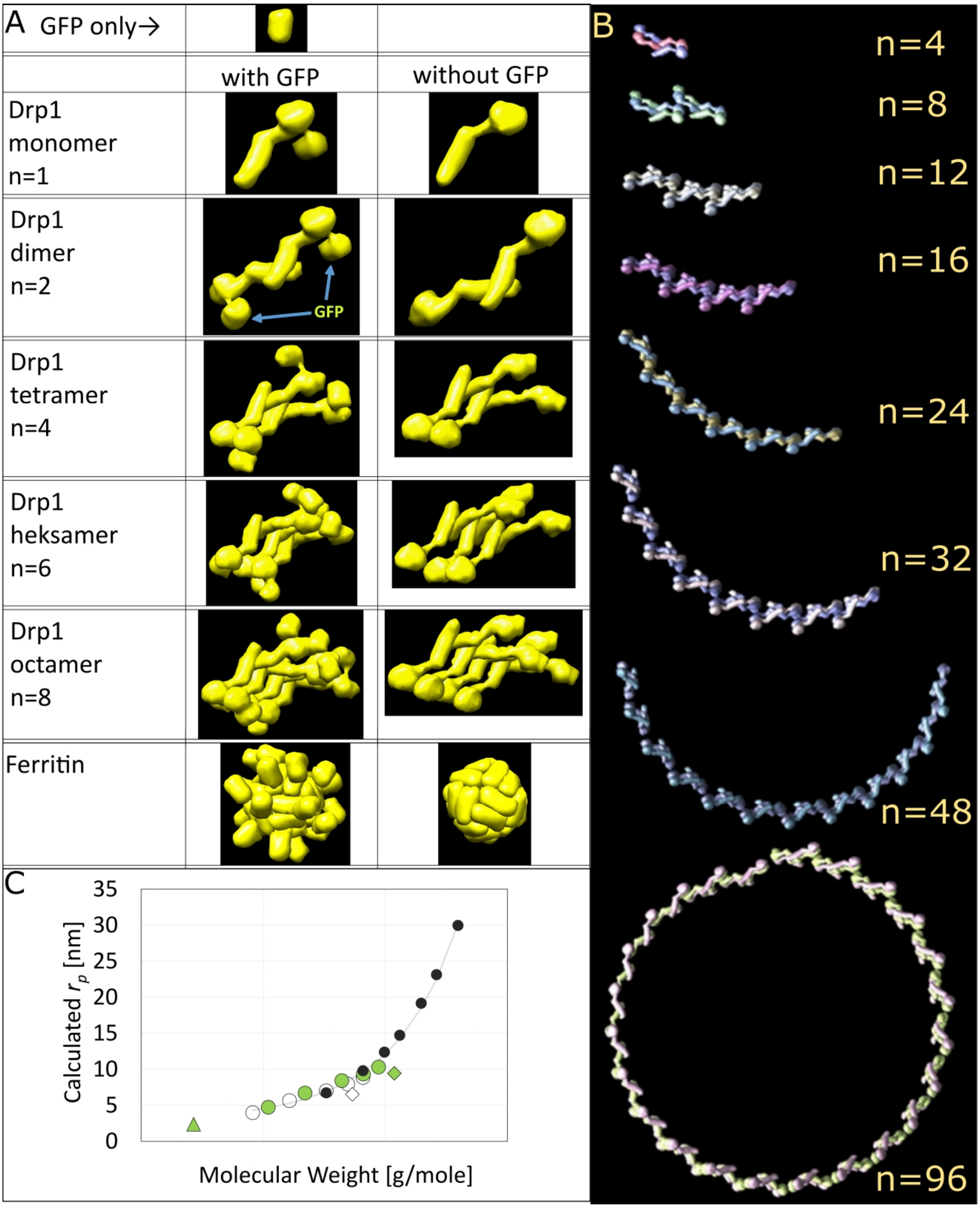

Supplementary Fig. S1. Sizes of the Drp1 oligomers. (A) The PDB structures of Drp1 (4BEJ), GFP (1EMA) and ferritin (4YKH) were used to build models of Drp1 and GFP-Drp1 oligomers as well as the model molecules (GFP, ferritin). The structure of GFP-Drp1 was created in Chimera software and saved in pdb format for calculations in HydroPro software. The four Drp1 molecules (monomers) in the asymmetric

unit of 4BEJ were used as a model of the tetramer. Biological assembly was used to obtain a model of the dimer, and one selected Drp1 molecule from a dimer was used as a model of the monomer. The hexamer and octamer were created by the addition of dimers to the tetramer model. (B) Larger Drp1 oligomers were obtained by manual fitting of the Drp1 dimer to the electron microscopy (EM) density map[22]. The obtained structure was divided into smaller fragments composed of 4, 8, 12, 16, 24, 32 and 48 Drp1 molecules. The largest structure used in calculation was the 48-mer due to the limitations of the HydroPro software. The obtained dependence of the size r_p on the molecular weight of the Drp1 oligomer is shown in (C). The calculated r_p values for GFP-Drp1 (green circles), Drp1 (empty circles), parts of the Drp1 ring (full black), GFP (green triangle), ferritin (empty diamond), and GFP-ferritin (full green diamond) are shown.

Supplementary Methods 1

Fluorescence correlation spectroscopy (FCS) measures the time series of fluorescence fluctuations originating from fluorescent molecules traveling through diffraction limited confocal spot of an optical setup. The autocorrelation of the obtained time-series fluctuations leads to autocorrelation function (ACF), which carries information concerning the concentration and diffusion of the fluorescent molecules[45,46]. Quantitative data can be obtained from this method after calibration of the system using a solution of a fluorescent dye with a known diffusion coefficient, which enables calculation of the parameters describing the confocal volume of the optical system. The confocal volume is described by a three-dimensional Gaussian intensity profile (equation S1),

$$I(x, y, z) = I_0 \exp\left(-\frac{2(x^2+y^2)}{w_0^2} - \frac{2z^2}{z_0^2}\right) \qquad \text{(S1)}$$

where I is the intensity of light, and w_0 and z_0 are the lateral and axial sizes of the beam waist.

FCS experiments were performed using a Leica SP8 commercial setup equipped with PicoQuant electronics for time-correlated single-photon counting (TCSPC) detection and single-photon avalanche diode (SPAD) detectors (external single-photon detectors) for single-molecule detection. The GFP fluorescence was collected using a 561 beam splitter and a 500-550 nm bandpass filter. Three spots were selected in each cell, and a single FCS curve was acquired for 30 seconds. The laser power was adjusted to collect approximately 10^4 photon counts per molecule for the Atto 488 Carboxy molecule, whose diffusion coefficient at 37 °C, given as 537 $\mu m^2 s^{-1}$, was used to calibrate the confocal volume (w_0=214 nm, z_0=1178 nm).

The autocorrelation curves were fitted and calculated using Fluctuation Analyzer software[47]. The two-component diffusion model, which assumes two types of molecular components (i=1,2) to be present in the solution, was used to fit the experimental data (equation S2),

$$G(\tau) = \frac{1}{N_a} H(\tau)\big(a_1 D_1(\tau) + (1 - a_1) D_2(\tau)\big) \qquad \text{(S2)}$$

where N_a is the sum of the number of molecules of both components (i=1,2) in the confocal volume, a_1 is the fraction of the first component, and $(1-a_1)$ is the fraction of the second component. $D_i(\tau)$, (i=1,2), represent terms describing the diffusion (equation S3),

$$D_i(\tau) = \frac{1}{1+\frac{\tau}{\tau_{D,i}}} \frac{1}{\sqrt{1+\frac{1}{\kappa^2}\frac{\tau}{\tau_{D,i}}}} \quad \text{(S3)}$$

where $\tau_{D,i} = w_0^2/(4D_i)$ is the diffusion correlation time for molecular component i, and $\kappa = w_0/z_0$ is the ratio of the lateral focal radius w_0 and its axial counterpart z_0 (κ=5.5 for our system). The obtained diffusion coefficients were calculated (equation S4) based on the measured diffusion correlation time, $\tau_{D,i}$,

$$D_i(\tau) = \frac{w_0^2}{4\tau_{D,i}} \quad \text{(S4)}$$

$H(\tau)$ is a term describing the photophysics of the fluorophore (equation 5),

$$H(\tau) = \left(1 - \theta_{nfl} + \theta_{nfl} exp\left(-\frac{\tau}{\tau_{nfl}}\right)\right) \quad \text{(S5)}$$

where θ_{nfl} and τ_{nfl} are the fraction and lifetime of the non-fluorescent (*nfl*) state (for GFP, we used τ_{nfl}=54 μs and θ_{nfl}=0.21). The resulting fit parameters were compared with the FCS model appropriate for the oligomerization reaction, in which species that differ in molecular brightness should be considered (equation S6)[48],

$$G(\tau) = \frac{1}{N_n} H(\tau) \left(f_1^2 D_1(\tau) + \frac{m}{n} f_1(1 - f_1) D_2(\tau)\right) \quad \text{(S6)}$$

where *n, m* denote the size of the oligomer given as the number of constituent monomers; N_n, N_m denote the number of oligomer molecules of size *n, m* in the confocal volume; and f_i denotes the contribution to total brightness from molecular component *i* (equation S7),

$$f_1 = \frac{nN_n}{N} = \frac{nN_n}{nN_n+mN_m}, f_2 = \frac{mN_m}{N} = \frac{mN_m}{nN_n+mN_m} \quad \text{(S7)}$$

where $N \times B$ is the total fluorescence signal obtained from a sample containing a single fluorophore of brightness B. The brightness B of the GFP fluorophore was estimated based on the FCS measurements performed on cells transfected with GFP or GFP-Drp1 K668E (GFP-Drp1 monomer). The value of B (B=4.4±0.3 kHz/s per molecule) was obtained as the slope of the linear dependence of the number of collected photons (using a SPAD detector) on the number of molecules in the confocal volume.

By comparing the terms in equations S2 and S6, one can obtain the following equation:

$$N_n = \frac{1}{N^2 a_1} N_a n^2 \qquad \text{(S8)}$$

which was used to calculate the concentration of GFP-Drp1 molecules in the cytoplasm.

The oligomeric form of Drp1 was estimated based on comparing the measured diffusion coefficients with the diffusion coefficients predicted based on the LDVM. The diffusion time obtained for the first molecular component ($i=1$) corresponds to the tetrameric form of GFP-Drp1 ($n=4$) and dominates the obtained autocorrelation curve. The second molecular component ($i=2$), with the diffusion coefficient of ~ 0.45±0.15 $\mu m^2 s^{-1}$, corresponds to a large Drp1 oligomer on the order of a 48-mer ($m=48$).

The concentration of tetrameric Drp1 was calculated as $c=N_n/V_{eff}$, (S9) using the estimated value of GFP-Drp1 tetramers in the confocal volume, N_4 (equation S8), and the effective volume of the confocal spot, $V_{eff} = \pi^{3/2} w_0^2 z_0$ (S10) (V_{eff}=0.3 fL). To calculate molar concentrations, N_n was divided by Avogadro's constant.

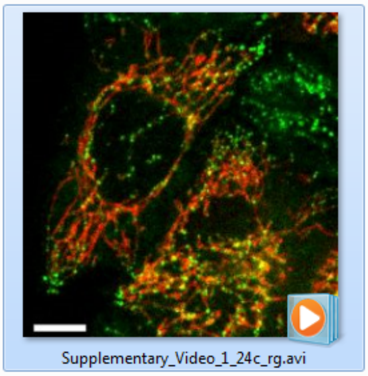

Supplementary Video 1. Time-lapse video of 24c. Total duration of the movie is 300 s (5 min) with the time step of 1 s. The size of the filed view is 60.11 by 62.01 μm. Video was saved in avi format using ImageJ, with JPG compression and frame rate of 20 frames per second (20 fps). Mitochondria are shown in red and the GFP-Drp1 is shown in green.

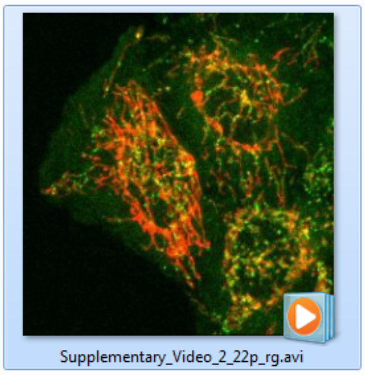

Supplementary Video 2. Time-lapse video of 22p. Total duration of the movie is 300 s (5 min) with the time step of 1 s. The size of the filed view is 69.63 by 72.38 μm. Video was saved in avi format using ImageJ, with JPG compression and frame rate of 20 frames per second (20 fps). Mitochondria are shown in red and the GFP-Drp1 is shown in green.

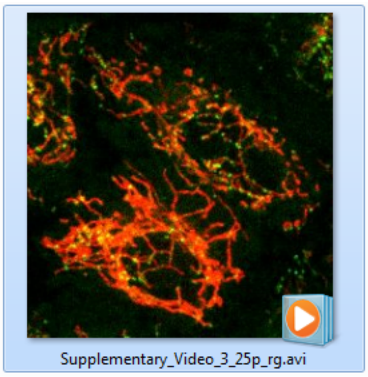

Supplementary Video 3. Time-lapse video of 25p. Total duration of the movie is 300 s (5 min) with the time step of 1 s. The image size is 63.92 by 68.15 μm. Video was saved in avi format using ImageJ, with JPG compression and frame rate of 20 frames per second (20 fps). Mitochondria are shown in red and the GFP-Drp1 is shown in green.

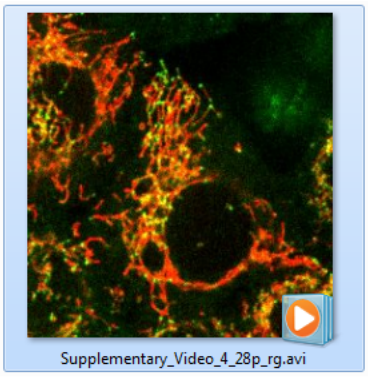

Supplementary Video 4. Time-lapse video of 28p. Total duration of the movie is 300 s (5 min) with the time step of 1 s. The image size is 63.92 by 68.15 μm. Video was saved in avi format using ImageJ, with JPG compression and frame rate of 20 frames per second (20 fps). Mitochondria are shown in red and the GFP-Drp1 is shown in green.

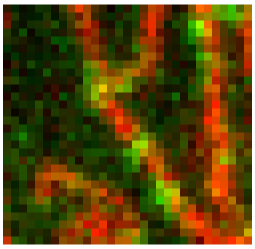

Supplementary Video 5. Time-lapse video of the 22p cell line showing successful fission event preceded by characteristic assembly phase (number of GFP-Drp1 molecules in the fission complex=173, Fig. 8A). Only the region of interest containing the fission event is shown. Total duration of the movie is 300 s (5 min) with the time step of 1 s. The image size is 6.56 by 6.35 μm. Video was saved in avi format using ImageJ, without compression and frame rate of 20 frames per second (20 fps). Mitochondria are shown in red and the GFP-Drp1 is shown in green.

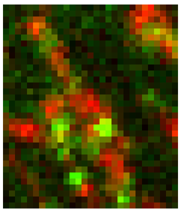

Supplementary Video 6. Time-lapse video of the 22p cell line showing successful fission event without characteristic assembly phase prior to actual fission event (number of GFP-Drp1 molecules in the fission complex=161, Supplementary Figure S2). Only the region of interest containing the fission event is shown. Total duration of the movie is 300 s (5 min) with the time step of 1 s. The image size is 6.14 by 7.20 μm. Video was saved in avi format using ImageJ, without compression and frame rate of 20 frames per second (20 fps). Mitochondria are shown in red and the GFP-Drp1 is shown in green.

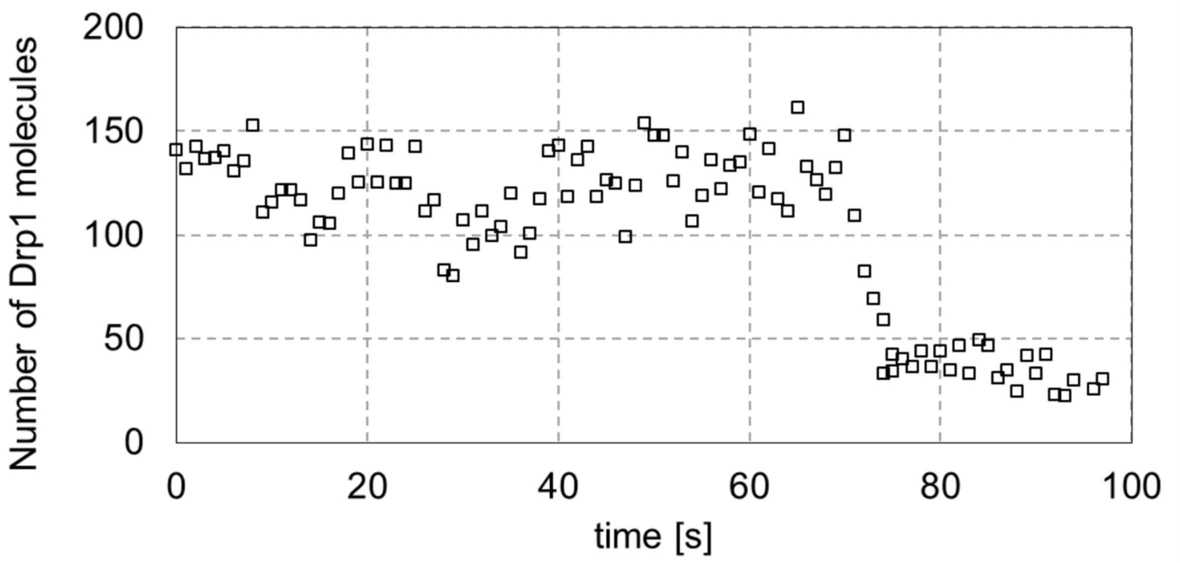

Supplementary Fig. S2 Time changes in the number of Drp1 molecules in the fission complex for the fission event presented on the Supplementary Video 6. The number of GFP-Drp1 molecules in the complex remains constant without clear assembly phase preceding the fission event (which occurs at the time point of around 70 s).

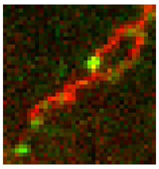

Supplementary Video 7. Time-lapse video of the 22p cell line showing successful fission event with fission complex consisting of 167 GFP-Drp1 molecules (Supplementary Fig. S3). The image size is 8.04 by 8.47 μm. Video was saved in avi format using ImageJ, without compression and frame rate of 20 frames per second (20 fps). Mitochondria are shown in red and the GFP-Drp1 is shown in green.

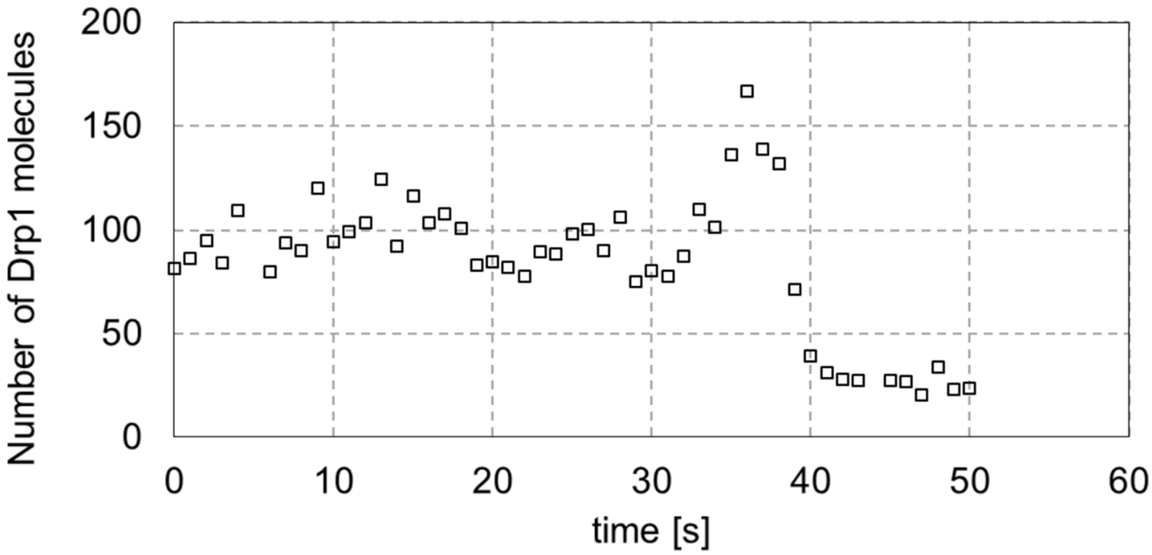

Supplementary Fig. S3 Example of fission event with the size of the fission complex surpassing the $N_{fission}$ threshold of 100 molecules. Number of Drp1 molecules in the fission complex as a function of time is shown for the fission event presented on the Supplementary Video 7. The number of GFP-Drp1 molecules in the complex increases from around 100 to around 170, when fission event occurs (at the time point of 36 s).

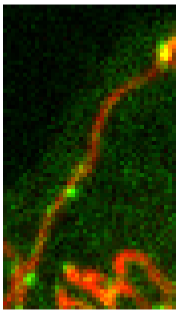

Supplementary Video 8. Time-lapse video of the 22p cell line showing fission complex composed of up to 110 GFP-Drp1 molecules (Supplementary Fig. S4). Although the number of GFP-Drp1 molecules surpasses the $N_{fission}$ number, the complex decomposes, and no fission is observed. The image size is 9.10 by 16.08 μm. Video was saved in avi format using ImageJ, without compression and frame rate of 20 frames per second (20 fps). Mitochondria are shown in red and the GFP-Drp1 is shown in green.

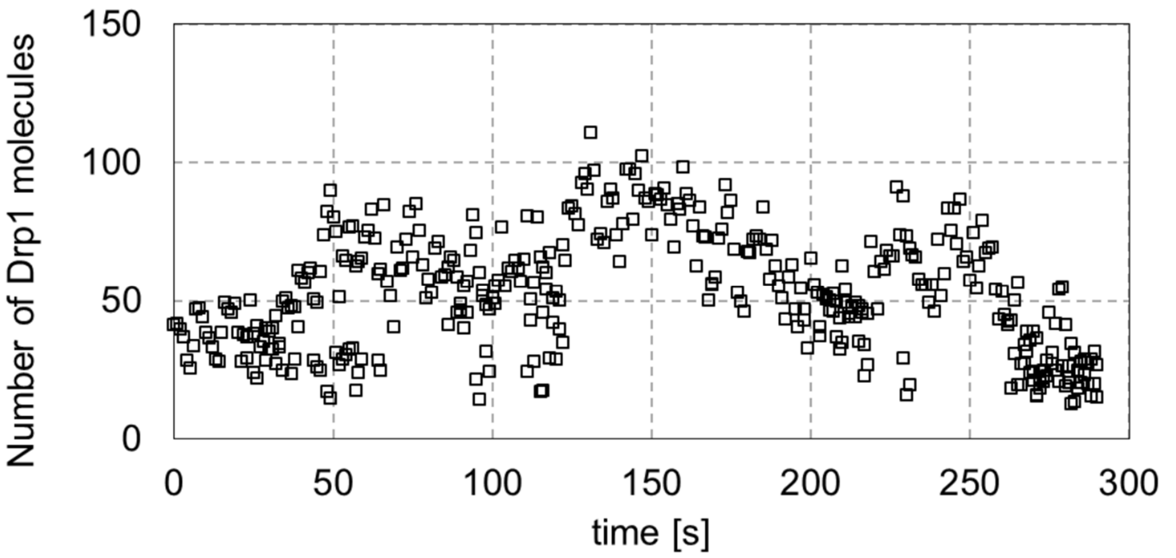

Supplementary Fig. S4 Changes in the number of Drp1 molecules in the fission complex for the fission event presented on the Supplementary Video 8. The number of GFP-Drp1 molecules in the complex reaches up to 110 molecules (around time of 130 s), however no fission event is observed.

molecule name	r_p [nm]	N	r_p [nm]
GFP	2.4	4	6.7
Drp1 monomer	4.0	8	9.8
Drp1 dimer	5.7	12	12.4
Drp1 tetramer	7.0	16	14.7
Drp1 hexamer	8.0	24	19.2
Drp1 octamer	8.9	32	23.1
GFP-Drp1 monomer	4.7	48	30.0
GFP-Drp1 dimer	6.7		
GFP-Drp1 tetramer	8.4		
GFP-Drp1 hexamer	9.3		
GFP-Drp1 octamer	10.3		
Ferritin	6.5		
GFP-ferritin	9.4		

Supplementary Table S1. Sizes, r_p, of the test probes and Drp1 oligomers derived from diffusion coefficients calculated using HydroPro software. The number of Drp1 molecules forming the oligomer derived from the ring-like structure (based on EM data) is denoted as *n*.

Supplementary Methods 2.

The basic assumption was that the histograms measured for the same cell lines should be similar when measured using different microscopes. The calibration curve used to translate the intensities, I_{SD}, obtained on a spinning disk (SD) microscope into the number of GFP-Drp1 molecules, N, was treated as a linear function, $I_{SD}(N)=a_{SD}\times N+b_{SD}$ (S11). The summary of the procedure used to obtain parameters a_{SD} and b_{SD} is as following. Step 1. The list of GFP-Drp1 spot intensities obtained using a confocal laser scanning microscope (LSM), I_{LSM}, was translated into the number of GFP-Drp1 molecules using the calibration function (Fig. 7A). The normalized histogram, $H_{LSM}(n)$, of the number of molecules, n, in the GFP-Drp1 spot was fit with the formula describing the oligomerization model (equation 4), giving the parameter Kc. Step 2. A normalized histogram, $H_{SD}(I_{SD})$, of the GFP-Drp1 spot intensities, I_{SD}, obtained using a SD microscope was made. Step 3. The histogram, $H_{SD}(I_{SD})$, was fit with the oligomerization model (equation 4), with the parameter Kc fixed to the value obtained in step 1. The $n-1$ exponent was expressed in terms of $I_{SD}(N)$, a_{SD} and b_{SD}, considering the tetrameric form of GFP-Drp1 as the oligomeric building block. The a_{SD} and b_{SD} parameters were obtained as free fit parameters with the following values: a_{SD}=125±14 and b_{SD}=3061±426. The measurements were performed using a Zeiss SD confocal microscope (Inverted Axio Observer Z.1 with environmental chamber). An EMCCD Evolve 512 camera was used for detection, with a resolution of 512x512, a 16 pixel bit depth and a pixel size of 16 μm×16 μm. The images were acquired with an HC APO 63x/1.20 Water immersion objective and usually contained several cells in a single field of view, with an image pixel size of 212 nm.

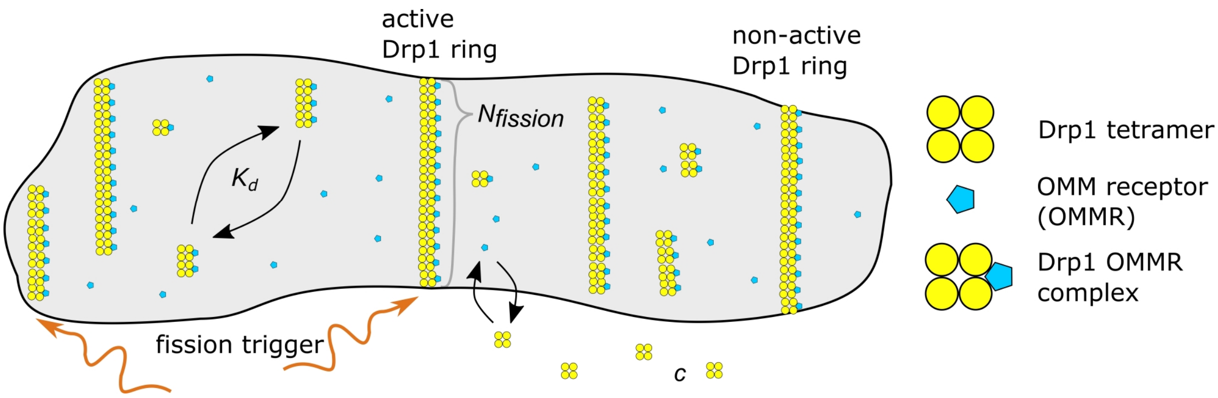

Supplementary Fig. S5. Model of Drp1 assembly at the mitochondria (gray). The predominant form of cytoplasmic GFP-Drp1 is a tetramer, present at a concentration, c, of 28 nM. The Drp1 tetramer can bind to its OMMR to form an OMMR-Drp1 complex. Such complexes can interact with each other with the dissociation constant K_d=31 nM, leading to the formation of OMMR-Drp1 oligomers. If the number of GFP-Drp1 molecules forming an oligomer surpasses the threshold of $N_{fission}$, the ring is susceptible to activation by a fission triggering signal (orange arrow). The number of fission events could be upregulated either by increasing the number of Drp1 rings surpassing the $N_{fission}$ threshold or by augmenting the frequency of fission triggering signals to activate more of the existing Drp1 rings.

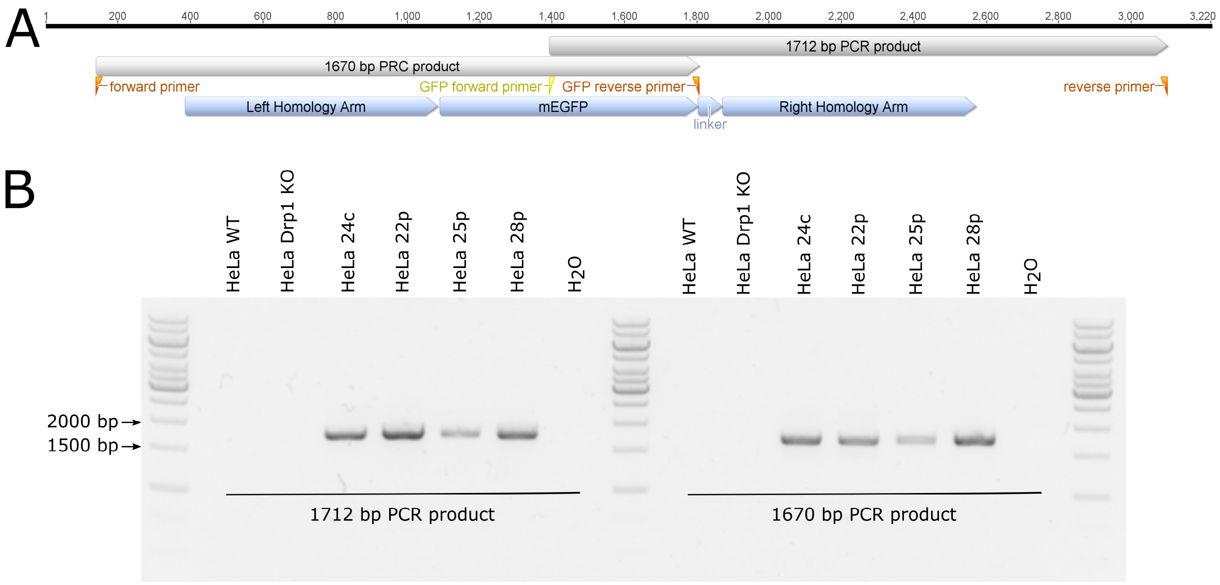

Supplementary Fig. S6. PCR products confirming incorporation of the GFP sequence in the appropriate place in the HeLa Kyoto genome (A) Fragment of DNM1L gene showing the localization of homology arms (sequences presented in donor vector plasmid), the GFP sequence, the primers used for PCR and the sizes of the PCR products. (B) Agarose gel electrophoresis showing PCR products obtained based on genomic DNA in HeLa Kyoto cell lines. The lack of bands (PCR products) in HeLa control samples (HeLa WT and HeLa Drp1 KO) indicates that the GFP sequence is not present in those cell lines. Original (non-inversed grayscale) scan of the agarose gel (in tif format) shown on the Supplementary Fig. S7.

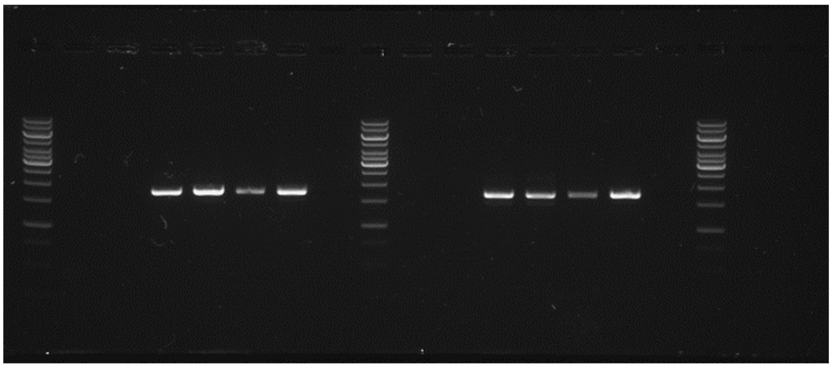

Supplementary Fig. S7. Image showing original scan of the agarose gel presented on Supplementary Fig. 6

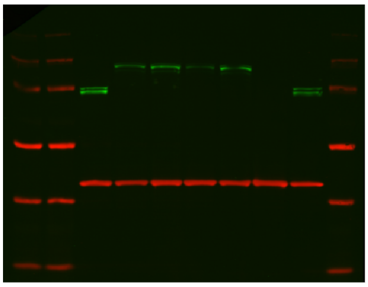
A

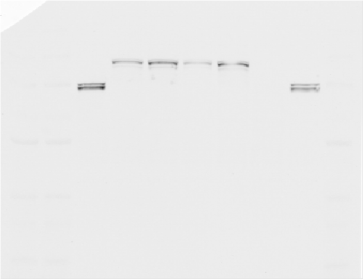
B

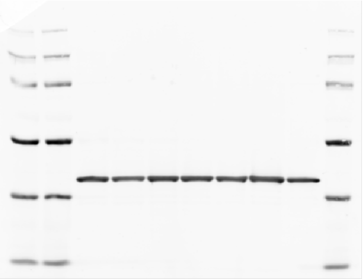
C

Supplementary Fig. S8. Scan (image in the tif format) of the western blot membrane for Drp1 and GAPDH obtained using Odyssey Infrared Imaging System (Li-Cor Biosciences, Lincoln, NE, USA). Drp1 (green color) was detected using primary mouse antibody and secondary donkey anti-mouse antibody labeled with infrared fluorescent dye IRDye 800CW (Li-Cor Biosciences) which fluorescence was collected in the 800 nm channel. GAPDH (red color) was detected with primary rabbit antibody and secondary donkey anti-rabbit antibody labeled with infrared fluorescent IRDdye 680LT (Li-Cor Biosciences) fluorescence was collected in the 700 nm channel. (A) Overlay of membrane scans for Drp1 (green) and GAPDH (red) (B) Drp1 signal in grayscale (C) GAPDH signal in grayscale. The following samples were loaded on a gel (wells from left to right): ladder, ladder, control (HeLa Kyoto WT), 24c, 22p, 25p, 28p, HeLa Kyoto Knock Out (KO), control (HeLa Kyoto WT). Fragment of gels containing lines from 3 to 8 were cropped and shown on the Fig. 5.

number of Drp1 isoform	RefSeq	Number of amino acids	Description
1	NP_036192	736 aa	This transcript has 20 exons and encodes isoform 1 (exon not used: 3). Canonical sequence for other isoforms
2	NP_036193	710 aa	This transcript has 19 exons and encodes isoform 2 (exons not used: 3, 16).
3	NP_005681	699 aa	This transcript has 18 exons and encodes isoform 3 (exons not used: 3, 16, 17).
4	NP_001265392	725 aa	This transcript has 19 exons and encodes isoform 4 (exons not used: 3, 17).
5	NP_001265393	749 aa	This transcript has 21 exons and encodes isoform 4 (all exons used).
6	NP_001265394	738 aa	This transcript has 20 exons and encodes isoform 6 (exon not used: 17).
7	NP_001265395	533 aa	This variant (7) contains an alternate exon in its 5' UTR, lacks four consecutive exons in the internal coding region, and initiates translation at an alternate start codon, compared to variant 1 (coding isoform 1). The encoded isoform (7) has a shorter and distinct N-terminus, compared to isoform 1
8	NP_001317309	712 aa	This transcript has 16 exons and encodes isoform 8 (exons not used: 16, 17).

Supplementary Table S2. Different isoforms of the protein Drp1. Information based on www.ensembl.org, for Human Gene DNM1L.
